# Spinal astrocyte dysfunction drives motor neuron loss in late-onset spinal muscular atrophy

**DOI:** 10.1007/s00401-023-02554-4

**Published:** 2023-03-17

**Authors:** Linda-Isabell Schmitt, Christina David, Rebecca Steffen, Stefanie Hezel, Andreas Roos, Ulrike Schara-Schmidt, Christoph Kleinschnitz, Markus Leo, Tim Hagenacker

**Affiliations:** 1 Department of Neurology, Center for Translational Neuro- and Behavioral Sciences (C-TNBS), University Hospital Essen, Hufelandstr. 55, 45147 Essen, Germany; 2 Department of Pediatrics 1, Division of Neuropediatrics, Center for Translational Neuro- and Behavioral Sciences (C-TNBS), University Hospital Essen, Hufelandstr. 55, 45147 Essen, Germany

**Keywords:** EAAT1, Excitotoxicity, Glutamate, Motor neuron disease, Arundic acid, Neuroprotection

## Abstract

Spinal muscular atrophy (SMA) is a progressive neuromuscular disorder caused by a loss of the *survival of motor neuron 1* (*SMN1*) gene, resulting in a loss of spinal motor neurons (MNs), leading to muscle weakness and wasting. The pathogenesis of MN loss in SMA and the selective vulnerability in different cellular populations are not fully understood. To investigate the role of spinal astrocytes in the pathogenesis of late-onset SMA, we used a mouse model in addition to in vitro approaches. Immunostaining, Western blot analysis, small interfering ribonucleic acid (siRNA) transfections, functional assays, enzyme-linked immunosorbent assay (ELISA), behavioral tests, and electrophysiological measurements were performed. Early activation of spinal astrocytes and a reduction of the excitatory amino acid transporter 1 (EAAT1) on postnatal day (P) 20 preceded the loss of spinal MNs in SMA mice occurring on P42. EAAT1 reduction resulted in elevated glutamate levels in the spinal cord of SMA mice at P20 and P42. SMA-like astrocytes generated by siRNA and an ex vivo model of glutamate excitotoxicity involving organotypic spinal cord slice cultures revealed the critical role of glutamate homeostasis in the degeneration of MNs. The pre-emptive administration of arundic acid (AA), as an inhibitor of astrocyte activation, to SMA mice prior to the loss of motor neurons (P28) resulted in elevated EAAT1 protein levels compared to vehicle-treated SMA mice and prevented the increase of glutamate in the spinal cord and the loss of spinal MNs. Furthermore, AA preserved motor functions during behavioral experiments, the electrophysiological properties, and muscle alteration of SMA mice. In a translational approach, we transfected healthy human fibroblasts with *SMN1* siRNA, resulting in reduced EAAT1 expression and reduced uptake but increased glutamate release. These findings were verified by detecting elevated glutamate levels and reduced levels of EAAT1 in cerebrospinal fluid of untreated SMA type 2 and 3 patients. In addition, glutamate was elevated in serum samples, while EAAT1 was not detectable. Our data give evidence for the crucial role of spinal astrocytes in the pathogenesis of late-onset SMA, a potential driving force for MN loss by glutamate excitotoxicity caused by EAAT1 reduction as an early pathophysiological event. Furthermore, our study introduces EAAT1 as a potential therapeutic target for additional SMN-independent therapy strategies to complement SMN-enhancing drugs.

**Supplementary Information:**

The online version contains supplementary material available at 10.1007/s00401-023-02554-4.

## Introduction

Spinal muscular atrophy (SMA) is a progressive neuromuscular disorder resulting in muscle wasting and weakness caused by the degeneration of motor neurons (MNs) in the ventral horn of the spinal cord. The SMA phenotype is classified based on the age of onset and its severity. All SMA subtypes are caused by homozygous deletion or compound heterozygous mutations in the *survival of motor neuron* (*SMN*) *1* gene, resulting in a lack of SMN protein [40, 42, 43, 69]. Besides *SMN1,* the SMN protein is encoded by the *SMN2* gene. *SMN2* differs from *SMN1* in a cytosine to thymine transition in exon 7, resulting in around 80–90% of truncated mRNA and subsequently in a non-functional protein. The *SMN2* copy number (determining the SMN level) is considered a major disease modifier for its negative correlation with disease severity [12, 39, 40].

Besides the *SMN1* defect, additional mechanisms contributing to SMA pathology are not yet fully understood. Current therapeutic strategies with nusinersen, risdiplam, or onasemnogene abeparvovec focus on enhancing the SMN protein level in spinal MNs. Those drugs are highly effective when the treatment starts early. Nevertheless, therapeutic efficacy varies when treated symptomatically, depending on phenotype and age at start of treatment [14, 19, 24, 46, 49–52]. In particular, individuals suffering from late-onset SMA only undergo therapy after long-standing MN loss; in such cases, restoration of SMN alone can only stop disease progression or restore motor function to a slight degree. Since the loss of spinal MNs is irreversible, other targets must be identified to develop new therapeutic strategies supporting current SMN-enhancing drugs.

Besides MNs, the SMN protein is widely expressed among other cell types across the CNS, such as spinal astrocytes. Astrocytes are the most common neuroglial cell type in the CNS and are elementary for the physiological functions of neurons, such as spinal motor neurons, so they are likely to contribute to the pathology of SMA. The importance of astrocytes within the pathogenesis of SMA has been demonstrated in mouse models of severe forms of SMA [1, 47]. While solely restoring SMN in MNs does not rescue the phenotype of the SMA mice, restoration in astrocytes or both cell types has been shown to significantly ameliorate the phenotype [58, 67].

One of the major functions of astrocytes is regulating and maintaining extracellular glutamate homeostasis by uptaking glutamate from the synaptic cleft and protecting neurons from excitotoxicity-mediated death [2, 4, 18, 35, 66]. For this, the proper function of the two glutamate transport proteins, excitatory amino acid transporter (*SLC1A3*, EAAT) 1 and 2, in astrocytes is crucial. We recently provided evidence for EAAT1 protein downregulation in the spinal cord of late-onset SMA mice and cultured SMA-like astrocytes generated by small interfering ribonucleic acid (siRNA) transfection [41], proposing a role for glutamate excitotoxicity as a potential mechanism for MN loss in the mild form of SMA.

Numerous studies have focused on the more severe forms of SMA, presenting a different and more rapid progressive pathology than the milder late-onset form. Nevertheless, investigating the potential pathology contributing to late-onset SMA is necessary to better understand SMA pathogenesis in general and recognize differences between the subtype-dependent mechanisms to enable new potential therapeutic targets complementing current treatment strategies to be identified.

However, the consequence of the EAAT1 downregulation or other astrocytic modulations, especially for the degeneration of the spinal MNs, has not yet been determined. A greater understanding of the interaction between astrocytes and MN and their role in SMA pathology can be a key to identifying further therapeutic targets to support current SMN-enhancing drugs.

Here, we investigated the role of EAAT1 in the pathogenesis of late-onset SMA in a translational approach using a mouse model, cell cultures of mouse or induced human astrocytes, and CSF or serum samples of SMA patients.

## Materials and methods

### Animals

SMN-deficient mice (FVB.Cg-*SMN1*^tm1Hung^Tg(*SMN2*)2Hung/J; SMA mice) were purchased from Jackson Laboratory (#005058, Bar Habor, ME, United States). These mice were homozygote for the murine *SMN1* knockout and the insert of human *SMN2* (four copies), reflecting late-onset SMA type, similar to human SMA type 2 and 3, with milder motor symptoms.

Mice were maintained and bred in the Animal Research Lab of the University Medicine Essen and were used for spinal cord tissue harvesting or in vivo experiments at different timepoints. Age-matched wild-type (wt) FVB/N mice served as control. Both sexes were used (no difference between sexes was observed). Animals from different litters were used for each experiment and were randomly picked. The animals were kept on a 12/12-h light/dark cycle with water and standard food pellets available ad libitum. Animals were monitored weekly to examine body condition, weight, and general health. When animals were included in in vivo experiments, they were monitored daily.

All experiments were conducted under the animal welfare guidelines of the University of Duisburg-Essen. Furthermore, the SMA mouse model used and in vivo experiments were approved by the State Agency for Nature, Environment and Consumer Protection (LANUV) in North Rhine-Westphalia, Germany (reference number 81–02.04–2020.A335; 81–02.03.2021.A078). The number of animals used for the experiments was in accordance with the 3Rs concept.

### Preparation of spinal cord and muscle sections

The spinal cord tissue of age-matched SMA and wt mice was removed by hydraulic extrusion. The lumbar part was separated, immediately snap-frozen in liquid nitrogen, and stored at − 80 °C until usage.

Next, 20 µm cryo-sections were prepared, and every fifth section of each spinal cord was mounted on an independent microscopy slide. Afterward, slides were used for immunostaining.

The *musculus tibialis anterior* was removed from SMA and wt mice to prepare muscle sections. Muscles were placed in a tissue mold with TissueTek, frozen in a mixture of isopropanol and dry ice, and stored at − 80 °C until usage. For immunostaining 20 µm longitudinal cryo-sections and for nicotinamide adenine dinucleotide hydrogen (NADH) staining, 12 µm cross sections were prepared.

### Spinal astrocyte cultures

Spinal astrocytes were isolated from wt mice, as described elsewhere [41]. In brief, animals were deeply anesthetized by isoflurane, and the spinal column was dissected. The entire spinal cord was removed by hydraulic extrusion and freed from the meninges to avoid fibroblast contamination. The lumbar part was separated, chopped into a slurry using a razor blade, and transferred to 0.25% trypsin/EDTA solution (#25200056, Thermo Fisher Scientific, Germany) for 30 min at 37 °C. Enzymatic digestion was stopped by adding DMEM/F12 (#210410202, Thermo Fisher Scientific, Germany) containing 10% fetal bovine serum (FBS, #16140071, Thermo Fisher Scientific, Germany). Afterward, spinal cord tissue was mechanically dissociated into a single-cell suspension. This suspension was filled to 10 ml using a cell culture medium containing DMEM/F12 supplemented with 10% FBS and 1% penicillin/streptomycin (P/S, #15140122, Thermo Fisher Scientific, Germany), placed in a 75-cm^2^ cell culture flask and incubated at 37 °C and 5% CO_2_. The medium was replaced with a fresh medium the next day and then every other day. When cells reached 65% confluence (10–14 days), flasks were shaken on an orbital shaker (250 rpm at 37 °C, 5% CO_2_) to remove microglia overnight. Afterward, the medium was replaced. Astrocytes were scraped from the flask bottom, counted, and placed on poly-d-lysine (PDL, Sigma-Aldrich, Germany)-treated glass coverslips in a 24-well plate (3500 cells per coverslip). After 7 days in vitro (DIV) post-replating, siRNA experiments were performed.

### Human skin biopsy samples

Informed consent was obtained from all patients. Furthermore, study approval was obtained from the University Duisburg-Essen ethics committee (approval number 19–9011-BO).

### Generation of induced human astrocytes from skin fibroblasts

Induced astrocytes were generated as described elsewhere [16]. Briefly, human fibroblasts from healthy donors were directly converted into induced neuronal progenitor cells (iNPCs) using retroviral vectors (Oct4; Sox2; Klf4; c-Myc #RF101, ALSTEM United States) in combination with neuralizing medium. After transfection, cells were maintained in conversion medium (DMEM/-F12, 1% GlutaMAX, 1% N-2 supplement, 1% B27-supplement, 1% P/S, 20 ng/mL human fibroblast growth factor (FGF)-basic (#100-18B PrepoTech, Germany), 20 ng/mL human epidermal growth factor (EGF; #AF-100–15 PreproTech, Germany), 5 µg/mL heparin) and replated (1:2) after 5 days. Cells were then replated again 1:2 on glass coverslips with PDL after 2–3 days and plated in iNPC medium (DMEM/F12, 1% GlutaMAX, 1% N-2 supplement, 1% B27-supplement, 1% P/S and 40 ng/ml human FGF-basic). Generated iNPCs were detached using accutase and then plated onto 24 wells plates, including PDL-coated glass coverslips. The iNPCs were then differentiated into astrocytes using an astrocytes conversion media (DMEM high glucose, 10% FBS, 1% P/S, and 0.2% N2-supplement) and cultivated until 80% confluence was reached.

### Transfection of cultured spinal astrocytes or induced human astrocytes

Transfection of cultured astrocytes to induced SMN-deficiency was performed at 7 DIV as described elsewhere [41]. Two hours before applying the siRNA (mouse *SMN1*, #SR408287, human SMN1, #SR304480 OriGene, United States) to the cells, the culture medium was replaced by FBS-free medium. Afterward, 10 nM of *SMN1* siRNA was mixed with 200 µM of Silence Mag (#SM11000, OZ Biosciences, France) and incubated for 15 min at room temperature (RT). Next, the cultured astrocytes were incubated with the complex for 2 h on a magnetic plate at 37 °C and 5% CO_2_. Finally, the magnetic plate was removed, and the cells were incubated until the next day when the siRNA medium was replaced by fresh culture medium. Astrocytes were kept in culture until they were used for experiments at 10 DIV.

### Cerebrospinal fluid and serum samples of SMA patients

CSF and serum samples of seven patients (age 23–66 years) with 5q-SMA (type 2 or 3) were analyzed. Samples were collected before (baseline) and after 6 months of treatment with SMN-enhancing drug nusinersen. CSF and serum samples were used for glutamate assay or enzyme-linked immunosorbent assay (ELISA). All patients gave written informed consent. Cerebrospinal fluid and serum from non-SMA patients served as control (diagnostic procedure to rule out CNS disease). Study approval was obtained from the University Duisburg-Essen ethics committee (approval number 18–8285-BO).

### Immunostaining

Lumbar spinal cord sections, spinal astrocyte, or induced human astrocyte cultures were fixed in 4% paraformaldehyde (PFA; in PBS, 15 min), washed, permeabilized (PBS, 0.1 v/w Triton X-100, 15 min), and blocked (PBS, 5% BSA, 1 h). For spinal cord sections and astrocyte cultures, primary antibodies for SMN (anti-rabbit, 1:200, #NBP2-763839, Novus Biologicals, Germany), neurofilament heavy polypeptide (SMI-32, anti-mouse, 1:500, #801701. Biolegend, USA), glial fibrillary acid protein (GFAP) (anti-mouse, 1:500, #63893, Sigma-Aldrich, Germany), or EAAT1 (anti-rabbit, 1:500, #250113, Synaptic Systems, Germany) were diluted in blocking solution and incubated overnight at 4 °C. Afterward, sections were washed and incubated with secondary antibodies (goat anti-mouse Cy3 1:300 or goat anti-rabbit Alexa Fluor 488, Dianova, Germany) and Dapi, for counterstaining of nucleus DNA, in blocking solution for 1.5 h at RT. Images were obtained using a Zeiss Axio Observer.Z1 Apotome fluorescence microscope, and Zeiss Zen software. All microscope settings, such as laser intensity, exposure time, or contrast, were kept constant for each protein to analyze.

Image J software (NIH) was used to measure immunoreactivity. Therefore, immunoreactivity-positive cells were selected using the free-hand tool. The fluorescence intensity of each protein was measured and normalized against the background area in each image. In addition, fluorescence intensity values in SMA mouse tissue were normalized to control wt tissue.

The number of motor neurons in the lumbar ventral horns of SMA mice spinal cord sections was calculated by counting SMI-32 positive cells and compared to the number in control wt mice. Only SMI-32 positive MNs with visible nuclei were counted. Three slices per animal were analyzed.

### Neuromuscular junction (NMJ) staining

M*.tibialis anterior* sections were incubated with primary antibody for presynaptic synaptophysin (anti-rabbit, 1:500, #101,203, Synaptic Systems, Germany) as described above. Afterward, sections were washed and incubated with a secondary antibody (goat anti-rabbit Cy3, 1:300, Dianova, Germany) in blocking solution for 1.5 h at RT. The postsynapse was additionally stained with α-bungarotoxin Alexa Fluor 488 (α-BTX, 1:500, #B13422, Thermo Fisher Scientific, Germany).

NMJ were analyzed using Image J software. The NMJ size was evaluated with Image J software. The immunoreactivity of presynaptic synaptophysin was measured as described above. Co-localization of presynaptic synaptophysin and postsynaptic α-BTX was analyzed using the co-localization tool.

### NADH staining of M. *tibialis anterior*

Cross sections of the M*. tibialis anterior* were incubated in NADH-tetrazolium solution (NADH, #N8129, Sigma Aldrich, Germany; Nitro blue tetrazolium, #N6876, Sigma Aldrich, Germany) for 30 min at 37 °C. The unbound solution was removed using successive washes with 30% acetone, 60% acetone, 90% acetone, 60% acetone, and 30% acetone and mounted with Immumount. Images were taken using a Zeiss Axio Observer.Z1 Apotome fluorescence microscope. The intensity of NADH staining and cross-sectional area were analyzed in Image J software by selecting each muscle fiber and recording integrated intensity and area. In addition, the number of type 1 (dark) and 2 (light) was counted.

### Western blot analysis

We performed Western blot analysis to substantiate the evaluated protein level by immunostaining. Therefore, the spinal cord tissue of SMA or wt mice or cell cultures were homogenized in RIPA buffer containing a protease inhibitor cocktail (Roche, Germany). The amount of protein in those lysates was determined by a bicinchoninic acid (BCA) protein assay.

Ten micrograms of protein were applied to 4–15% TGX Stain-Free gels (Biorad, Germany), and proteins were transferred to 0.2 µm nitrocellulose membranes using a semi-dry blotting technique. Images of membranes were taken for total-protein evaluation. Afterward, the membranes were incubated in fast-blocking solution (Biorad, Germany) under gentle agitation for 10 min at RT. Then, the membranes were incubated with primary antibodies (in blocking solution) against SMN (anti-rabbit, 1:10,000, #NBP2-763839, Novus Biologicals, Germany), GFAP (anti-mouse, 1:5000, #63893, Sigma-Aldrich, Germany), or EAAT1 (anti-rabbit, 1:5000, #250113, Synaptic Systems, Germany) at 4 °C overnight. Primary antibody against β-actin was used as an additional loading control.

After washing, the membranes were incubated with anti-rabbit or anti-mouse horseradish peroxidase-coupled secondary antibody for 1.5 h at RT. Immunoreactivity was detected using an enhanced chemiluminescence substrate and a Western blot imaging system (Biorad, Germany).

Analysis of Western blot signals was performed using Biorad imaging software. First, the signal of each protein and actin lane was measured. Then, the protein signal of each lane was normalized to its total protein value. Finally, the calculated protein level of SMN mice was further normalized to the value detected in age-matched wt mice.

For detecting different proteins on the same membrane, those were stripped, blocked, and antibodies were incubated, as described above.

All Western blots and total protein staining used for mean value calculations are provided in the supplementary material (Supplementary material Figs. 1–6, online resource).

### Ribonucleic acid (RNA) isolation and real-time polymerase chain reaction (qPCR)

Total RNA was extracted from spinal cord samples or cell cultures using Qiazol (Qiagen #79306). One microgram of each RNA sample was used for first-strand complementary deoxyribonucleic acid (cDNA) synthesis in a 20-μl reaction with high-capacity cDNA RT Kit (Applied Biosystems #4368814). The expression levels of EAAT1 (forward GCGATTGGTCGCGGTGATAATG, reverse CGACAATGACTGTCACGGTGTAC; #MP215637, OriGene, United States) were quantitated by a real-time qPCR analysis using Power SYBR™ Green PCR Master Mix (#4,367,659, Applied Biosystems, United States). Data were normalized to the housekeeping gene β-Actin (forward CATTGCTGACAGGATGCAGAAGG, reverse TGCTGGAAGGTGGACAGTGAGG; # MP200232, OriGene, United States).

### Ex vivo model of glutamate excitotoxicity

Organotypic spinal cord slice cultures (OTSCs) were prepared from wt mice. Spinal cords were cut into 350 µm sections using a vibratome (Leica, Germany) and slices were placed on membrane inserts (0.4 µm pore size, Sarstedt, Germany) in six-well plates containing 1 ml of culture medium (Neurobasal A, 1% P/S, 1:50 B27). After culturing for 48 h, slices were exposed to glutamate (50 or 500 µM) or 0.9% saline (control) for 1 h at 37 °C and 5% CO_2_. This was followed by replacement of the medium to glutamate-free medium, and slices were incubated for an additional 48 h. Alternatively, spinal cord slices were exposed to PDC (100 or 200 µM) or 0.9% saline (control) for 48 h.

Finally, OTSCs were fixed and stained for SMI-32 as a marker for spinal MNs. The number of MNs in the ventral horn was counted and compared between the two conditions as described above. In addition, slices and supernatants were used for glutamate assay.

### Glutamate assay

The glutamate level in the spinal cord tissue of wt and SMA mice was measured by preparing a tissue lysate at different timepoints of SMA pathology. The tissue of age-matched wt mice served as control.

To measure glutamate uptake in SMN-deficient cultured spinal astrocytes or induced human astrocytes, wt astrocytes, or wt astrocytes incubated with EAAT inhibitor L-*trans*-pyrrolidine-2,4-dicarboxylic acid (PDC, Tocris, UK), 200 µM glutamate was applied to the cultured cells at 10 DIV. After 4 h of exposure to glutamate, the astrocyte-conditioned medium was collected.

According to the manufacturer’s protocol, the glutamate level of the collected astrocyte-conditioned medium, slice culture supernatant, the spinal cord tissue lysate or human CSF, and serum samples was evaluated using a glutamate assay kit (#MAK004, Sigma Aldrich, USA). The total protein amount in each sample was determined using a BCA protein assay. Afterward, the measured amount of glutamate was normalized to the amount of total protein in the corresponding sample. The calculated values for the SMN samples were normalized to the corresponding controls.

### ELISA

To determine the EAAT1 level in CSF or serum samples of SMA patients and healthy control individuals, an ELISA assay was performed according to the manufacturer’s protocol (#MBS070849, MyBioSource, USA).

### In vivo treatment of SMA and wt mice

Arundic acid (AA; 4 mg/kg BW; Caymanchem, USA) was diluted in PBS + 0.5% dimethyl sulfoxide (DMSO; Sigma-Aldrich, Germany) and administered daily via intraperitoneal (i.p.) injection. Mice were either treated with AA or vehicle (PBS + 0.5% DMSO) from P25 to P44. The motor behavior of the mice was tested at P33 and P44. Finally, the animals were sacrificed for tissue removal at P44. Experimentators were blinded for treatment, behavior testing, immunostaining, and analysis.

### Motor behavior testing

#### Grip strength

A grip strength meter (Ugo Basil, Italy) was used to measure hindlimb grip strength. In brief, the animal was placed on an angled mesh facing away from the meter. The tail was pulled toward the meter parallel to the mesh until the hindlimbs were released. Three strength measurements were recorded consecutively (within 2 min). Finally, the mean value of all the trials was calculated.

#### Rotarod

To assess motor coordination and balance, the rotarod running time was recorded. Mice were placed on a rotarod with a start speed of four rounds per minute (rpm) and an acceleration rate of 40 rpm/min, with a maximum cutoff time of 300 s. Three runs per animal were recorded, and the mean value was calculated.

#### Nerve conduction studies (NCS)

Recordings of the compound muscle action potential (CMAP) were used to identify changes within the motor nerve conduction. The animals were anesthetized by isoflurane/O_2_ inhalation (for initiation of anesthesia 5%, for maintenance 2% isoflurane in 100% oxygen). After the loss of measurable pain reflexes, the sciatic nerve was first stimulated at the sciatic notch using microneedle electrodes before stimulating at the ankle. Finally, the reference electrode was placed at the nape of the neck. A single pulse was delivered each time using 5 Hz for 0.1 ms. The subtracted latency of both measurements, as well as the amplitude, was recorded.

### Statistical analysis

Experimental results were statistically analyzed using Graph Pad Prism 9.4.1 software. First, all data were checked for normal distribution using the Shapiro–Wilk test. Then, depending on the experiment, data were analyzed using Student’s *t* test (Welch’s *t* test; two groups; normally distributed), Mann–Whitney *U* test (two groups; not normally distributed), ordinary one-way ANOVA with Holm–Sidak’s multiple comparison test (three groups; normally distributed or Kruskal–Wallis test with Dunn’s multiple comparison test (three groups; not normally distributed).

SMA tissue or SMA-like cells were always compared to aged-matched wt tissue or control cells at defined timepoints. The actual test used for each experiment can be found in the supplementary material (supplementary material, Table 1, online resources).

Levels of significance were defined according to the *p* value (*p < 0.05, **p < 0.01, or ***p < 0.001). All values were given as mean ± standard error of mean (SEM).

## Results

### Late-onset SMA mouse model reflects slow disease progression in mildly affected SMA patients

**Fig. 1:**
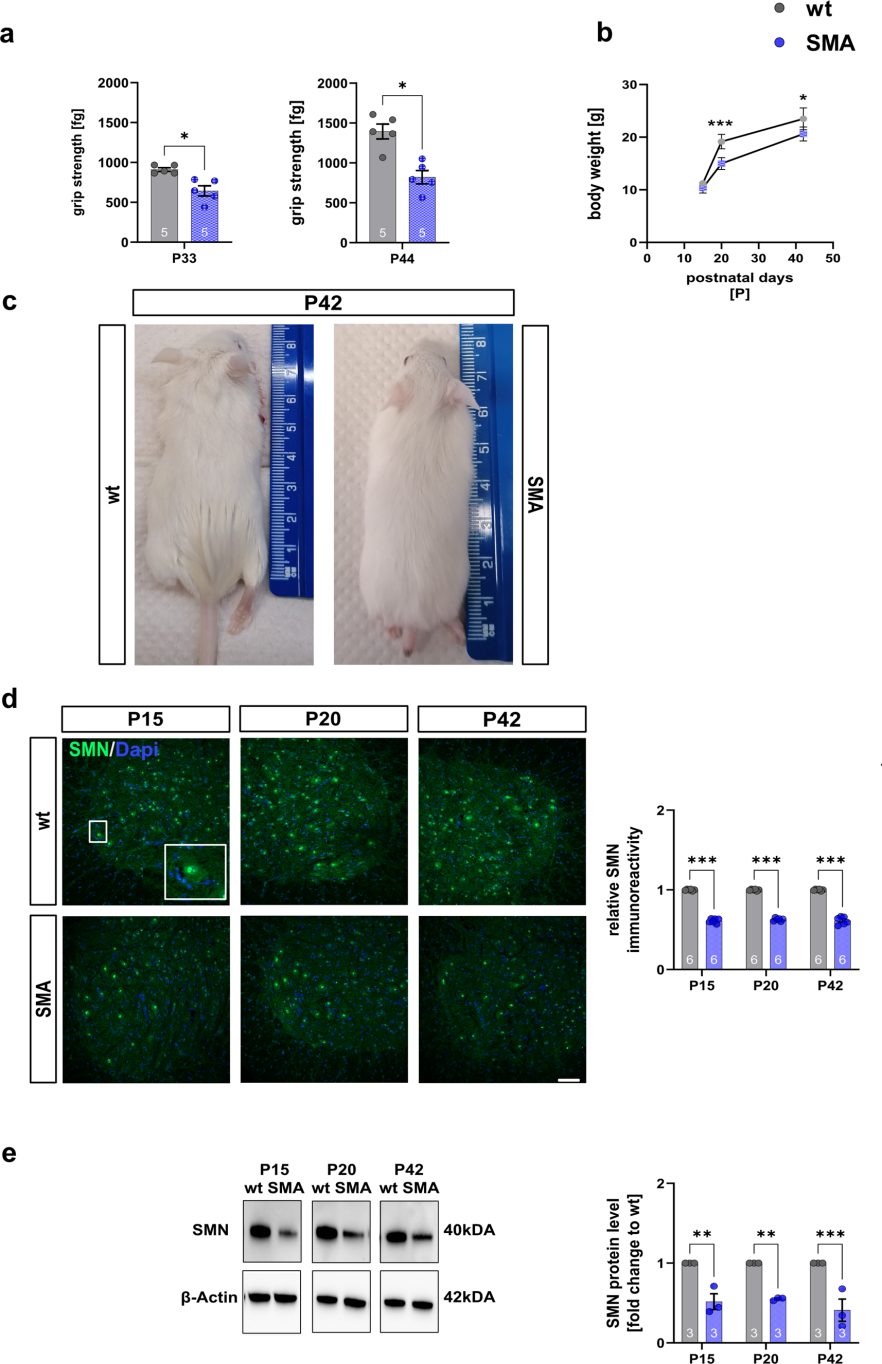
Late-onset SMA mice show phenotypical changes, reduced body weights, and SMN protein levels. **a** SMA mice showed reduced grip strength at P33 (*p* < 0.05) and P44 (*p* < 0.05). **b** Body weight in SMA mice was reduced at P20 and P42 compared to wt mice (*p* < 0.001). **c** Photographs of wt and SMA mice at P42 showing necrotic tail tissue under SMA conditions at P42. At P15, no difference was observed (*p* > 0.05). **d** Immunostaining of SMN (green) in the ventral horn of lumbar spinal cord slices from wt or SMN mice at P15, P20, and P42. Nucleic DNA was stained with Dapi (blue). The relative SMN level in the ventral horn of SMA mice spinal cords was reduced at all timepoints compared to wt mice (*p* < 0.001). **e** Western blot analysis of SMN protein levels in spinal cord tissue of wt or SMA mice at P15, P20, and P42. Beta-actin was used as a loading control. Analysis revealed reduced SMN protein levels in SMA mice at all timepoints compared to wt control mice (*p* < 0.01 to *p* < 0.001). *n* = 6 animals per condition for immunostaining. Three slices per lumbar spinal cord were investigated. Each data point reflects the mean of three spinal cord slices per animal. *n* = 3 animals per condition for Western blot analysis. *n* = 12 animals per condition for body weight analysis. Scale bar: 20 µm. Abbreviations: P, postnatal day; SMA, spinal muscular atrophy; SMN, survival of motor neuron; wt, wild type. *P* values: *p < 0.05, **p < 0.01, or ***p < 0.001

Late-onset SMA mice demonstrated reduced grip strength compared to wt mice as a sign of mild hindlimb paresis at P33 (*p* = 0.031) and P44 (*p* = 0.028) (Fig. 1a).

In addition, SMA mice showed reduced body weight at P20 (*p* = 0.0007) and P42 (*p* = 0.032) (Fig. 1b). Phenotypic changes were observed in late-onset SMA mice during their development. The animals showed signs of tail necrosis, resulting in tissue loss and shorter tails, compared to wt mice of the same age (Fig. 1c).

In immunostaining and Western blot analysis of lumbar spinal cord tissue, a lower SMN protein level was detected at all investigated timepoints (P15, P20, P42: *p* < 0.0001; Fig. 1d) (P15: *p* = 0.078; P20: *p* = 0.063; P42: *p* = 0.0001; Fig. 1e) (Supplementary Fig. 1, online resource).

### Increased reactivity of astrocytes precedes the loss of spinal MNs

**Fig. 2:**
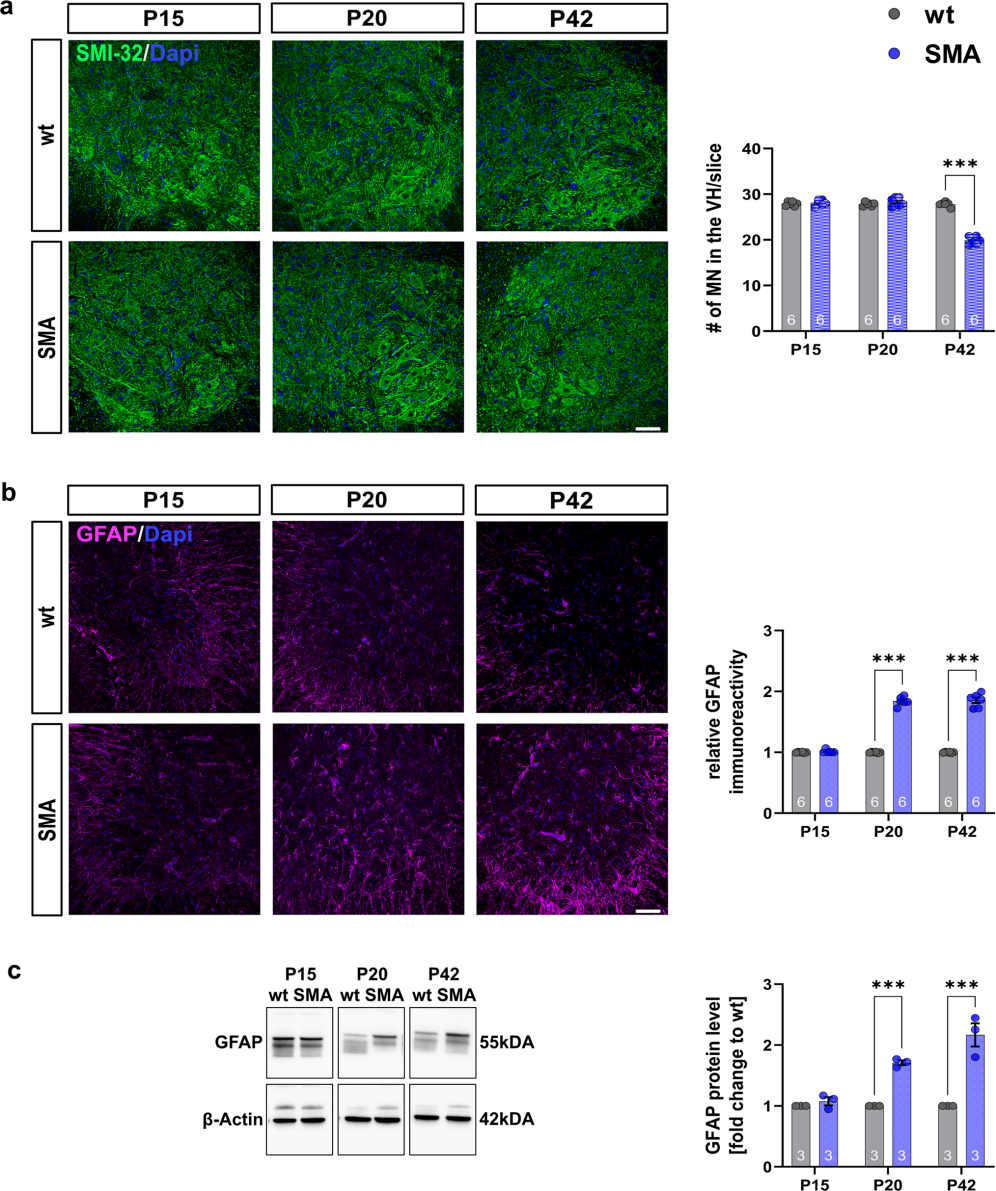
Spinal astrocyte activation precedes the loss of spinal MN.** a** Immunostaining of spinal MN (SMI-32, green) in the ventral horn of lumbar spinal cord slices from wt or SMA mice at P15, P20, and P42. Nucleic DNA was stained with Dapi (blue). The number of spinal MNs in SMA mice was reduced at P42 (*p* < 0.001). **b** Immunostaining of GFAP (magenta) as a marker of astrocyte reactivity in the ventral horn of lumbar spinal cord slices from wt or SMA mice at P15, P20, and P42. Nucleic DNA was stained with Dapi (blue). SMA mice showed an elevated relative GFAP protein level at P20, before MN loss, and P42 compared to wt mice (*p* < 0.001). **c** Western blot analysis of GFAP protein levels in spinal cord tissue of wt or SMA mice at P15, P20, and P42. Beta-actin was used as a loading control. SMA mice showed an enhanced GFAP protein level at P20 and P42 compared to wt mice (*p* < 0.001). *n* = 6 animals per condition for immunostaining. Three slices per lumbar spinal cord were investigated. Each data point reflects the mean of three spinal cord slices per animal. *n* = 3 animals per condition for Western blot analysis. Scale bar: 20 µm. Abbreviations: GFAP, glial fibrillary acid protein; MN, motor neuron; P, postnatal day; SMA, spinal muscular atrophy; wt, wild type; #, number. *P* values: *p < 0.05, **p < 0.01, or ***p < 0.001

Immunostaining for MN marker SMI-32 revealed the first loss of spinal MNs in SMA mice at P42 (*p* < 0.001), while no change was observed at P15 or P20 (P15: *p* = 0.522; P20:* p* = 0.233) (Fig. 2a. Supplementary Fig. 7, online resource).

Immunostaining and Western blot analysis for GFAP indicated an astrocytic reactivity by increased GFAP protein levels as a marker of activation of spinal astrocytes in SMA mice at P20 (*p* < 0.0001) before the loss of spinal MNs, and at P42 (Fig. 2b, c. Supplementary Fig. 2, online resource).

### Reduction of EAAT1 and increased glutamate level as a driving force for MN loss

**Fig. 3:**
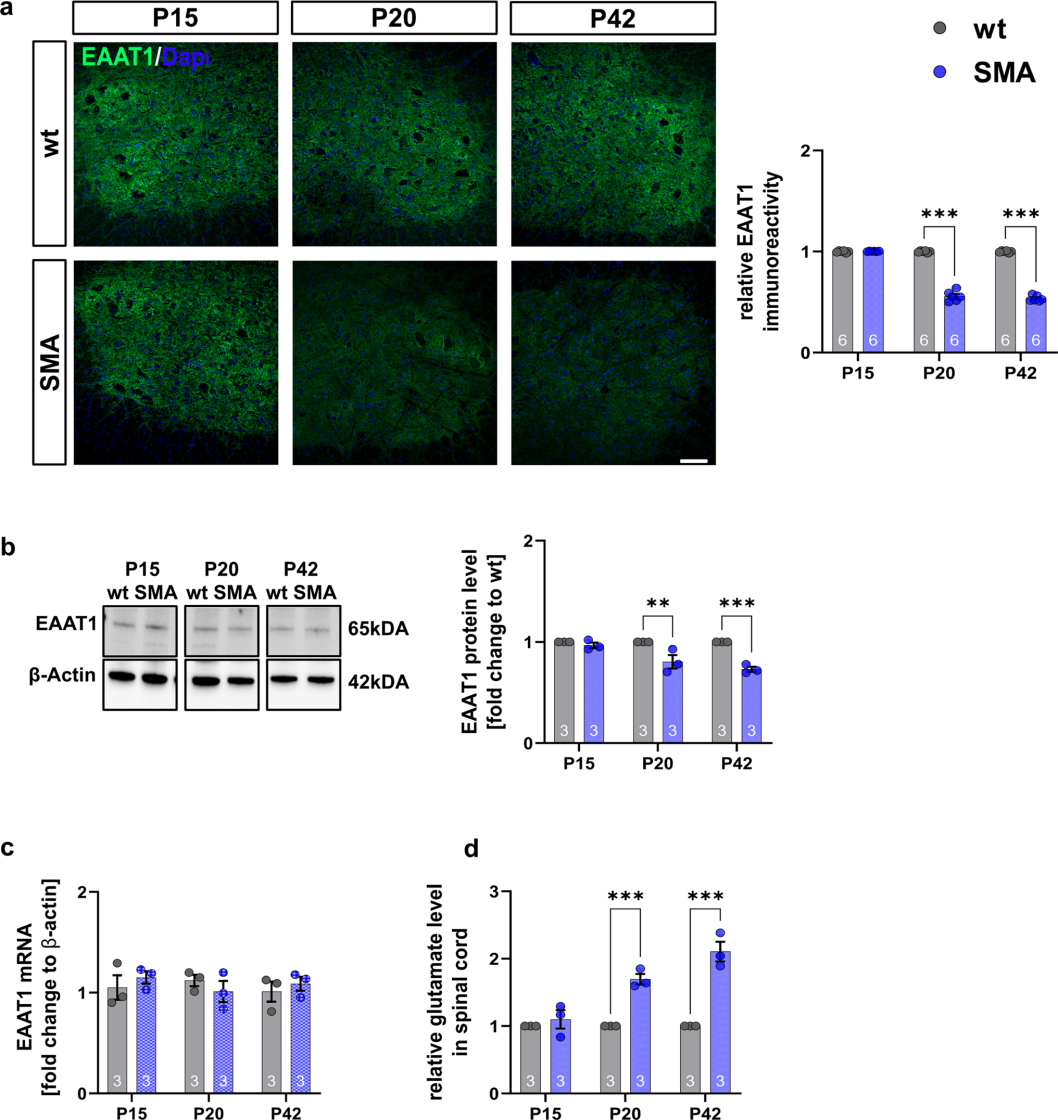
Late-onset SMA mice show early reduced EAAT1 expression and increased glutamate levels. **a** Immunostaining of EAAT1 (green) in the ventral horn of lumbar spinal cord slices from wt or SMA mice at P15, P20, and P42. Nucleic DNA was stained with Dapi (blue). The relative EAAT1 protein level in the ventral horn of SMA mice was reduced at P20, before motor neuron loss, and P42 (*p* < 0.001) compared to wt mice. **b** Western blot analysis of EAAT1 protein levels in spinal cord tissue of wt or SMA mice at P15, P20, and P42. Beta-actin was used as a loading control. SMA mice showed a reduced EAAT1 protein level at P20 (*p* < 0.01) and P42 (*p* < 0.001) compared to wt mice. **c** No change of EAAT1 mRNA expression was observed at any timepoint (*p* > 0.05). **d** Measurement of glutamate level in the spinal cord of wt or SMA mice at P15, P20, and P42 using a glutamate assay kit. The glutamate level in the spinal cord tissue of SMA mice was elevated at P20 (*p* < 0.001) and P42 (*p* < 0.001) compared to wt mice. *n* = 6 animals per condition for immunostaining. Three slices per lumbar spinal cord were investigated. Each data point reflects the mean of three spinal cord slices per animal. *n* = 3 animals per condition for Western blot analysis, qPCR analysis, and glutamate measurements. Scale bar: 20 µm. Abbreviations: EAAT1, excitatory amino acid transporter 1; mRNA, messenger ribonucleic acid; P, postnatal day; qPCR, real-time polymerase chain reaction; SMA, spinal muscular atrophy; wt, wild type. *P* values: *p < 0.05, **p < 0.01, or ***p < 0.001

EAAT1 protein levels were reduced in SMA mice from P20 (P20: *p* = 0.0009; P42: *p* < 0.0001, Fig. 3a; P20: *p* = 0.003; P42:* p* = 0.0005, Fig. 3b) (Supplementary Fig. 3, online resource). In contrast, EAAT1 mRNA levels were not altered (*p* > 0.05) in SMA mice (Fig. 3c). Glutamate levels were elevated at P20 (*p* < 0.001) and P42 (*p* < 0.001) (Fig. 3d), demonstrating a correlation between EAAT1 protein reduction and elevated glutamate levels.

**Fig. 4:**
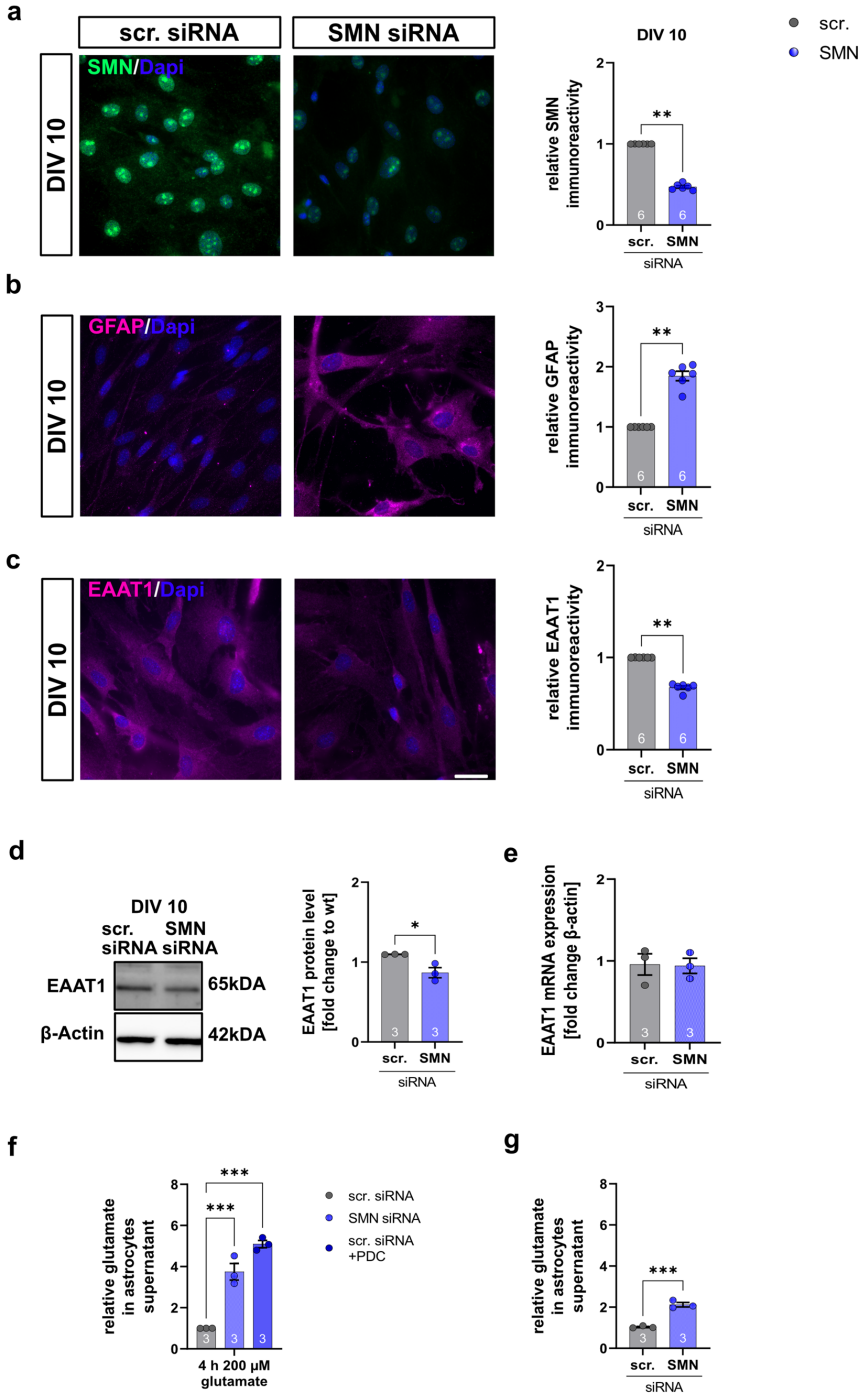
SMA-like astrocytes generated by siRNA show similar protein alteration and dysfunctions in glutamate uptake as the late-onset SMA mouse model.** a** Immunostaining of SMN (green) in cultured wt spinal astrocytes transfected with scrambled or *SMN1* siRNA at DIV 10. Nucleic DNA was stained with Dapi (blue). Astrocytes transfected with SMN siRNA showed reduced SMN protein levels (*p* < 0.01). **b** Immunostaining of GFAP (magenta) in cultured wt spinal astrocytes transfected with scrambled or SMN siRNA at DIV 10. Nucleic DNA was stained with Dapi (blue). Astrocytes transfected with SMN siRNA showed elevated GFAP protein levels compared to astrocytes transfected with scrambled siRNA (*p* < 0.01). **c** Immunostaining of EAAT1 (magenta) in cultured wt spinal astrocytes transfected with scrambled or SMN siRNA at DIV 10. Nucleic DNA was stained with Dapi (blue). Astrocytes transfected with SMN siRNA showed reduced EAAT1 protein levels compared to scrambled siRNA-transfected cells (*p* < 0.01). **d** In Western blot analysis the EAAT1 protein level in SMA-like astrocytes was reduced (*p* < 0.05) **e** No alteration in EAAT mRNA expression was detected (*p* > 0.05) in qPCR analysis. **f** When scrambled or SMN siRNA transfected astrocytes were exposed to 200 µM glutamate for 4 h, SMN-deficient astrocytes showed reduced glutamate uptake (*p* < 0.001). A similar effect was observed when scrambled siRNA-transfected astrocytes were exposed to the EAAT1 inhibitor PDC. Glutamate uptake was reduced compared to cells not exposed to PDC (*p* < 0.001). **g** Astrocytes transfected with SMN siRNA but not exposed to glutamate showed an increased release of glutamate compared to astrocytes transfected with scrambled siRNA (*p* < 0.001). *n* = 6 independent experiments for immunostaining. For each experiment, > 50 cells were analyzed per condition. *N* = 3 independent experiments for functional assays, qPCR, and Western blot analysis. Scale bar: 50 µm. Abbreviations: DIV, days in vitro; EAAT1, excitatory amino acid transporter 1; GFAP, glial fibrillary acid protein; mRNA, messenger ribonucleic acid; PDC, L-*trans*-pyrrolidine-2,4-dicarboxylic acid; qPCR, real-time polymerase chain reaction; siRNA, small interfering ribonucleic acid; SMN, survival of motor neuron; wt, wild type. *P* values: *p < 0.05, **p < 0.01, or ***p < 0.001

To induce SMN deficiency and generate SMA-like cells, cultured spinal astrocytes or cultured induced human astrocytes were transfected with scrambled (control) or *SMN1* siRNA using magnetic transfection. In *SMN* siRNA transfected cultured wt spinal astrocytes (SMA-like astrocytes), a reduction of the SMN protein level was detected by immunostaining (*p* = 0.002) (Fig. 4a). Immunostaining and Western blot analysis revealed a reduction of EAAT1 protein level (*p* < 0.002, Fig. 4c; *p* < 0.0022, Fig. 4d) (Supplementary fig, 6, online resource) in SMA-like astrocytes. No alteration in EAAT1 mRNA level (*p* > 0.999) was detected (Fig. 4e). As in the slice cultures, GFAP levels were elevated in SMN-deficient astrocytes (*p* = 0.002) (Fig. 4b).

To measure glutamate uptake, SMA-like astrocytes or scrambled siRNA transfected control cells, astrocytes were exposed to 200 µM of glutamate for 4 h, and the glutamate level in the supernatant was measured. In SMA-like astrocytes, glutamate uptake was reduced (*p* < 0.001).

Modulating the glutamate uptake in cultured wt spinal astrocytes with PDC as an EAAT1 inhibitor reduced the glutamate uptake from the medium (*p* < 0.0001), indicating the importance of EAAT1 for glutamate homeostasis (Fig. 4f).

Even without glutamate exposition, supernatant glutamate levels of SMA-like astrocytes were increased compared to scrambled siRNA transfected control cells, demonstrating the release of this transmitter of astrocytic origin (*p* < 0.0001) (Fig. 4g).

### Death of spinal MNs in an ex vivo model of glutamate excitotoxicity

**Fig. 5:**
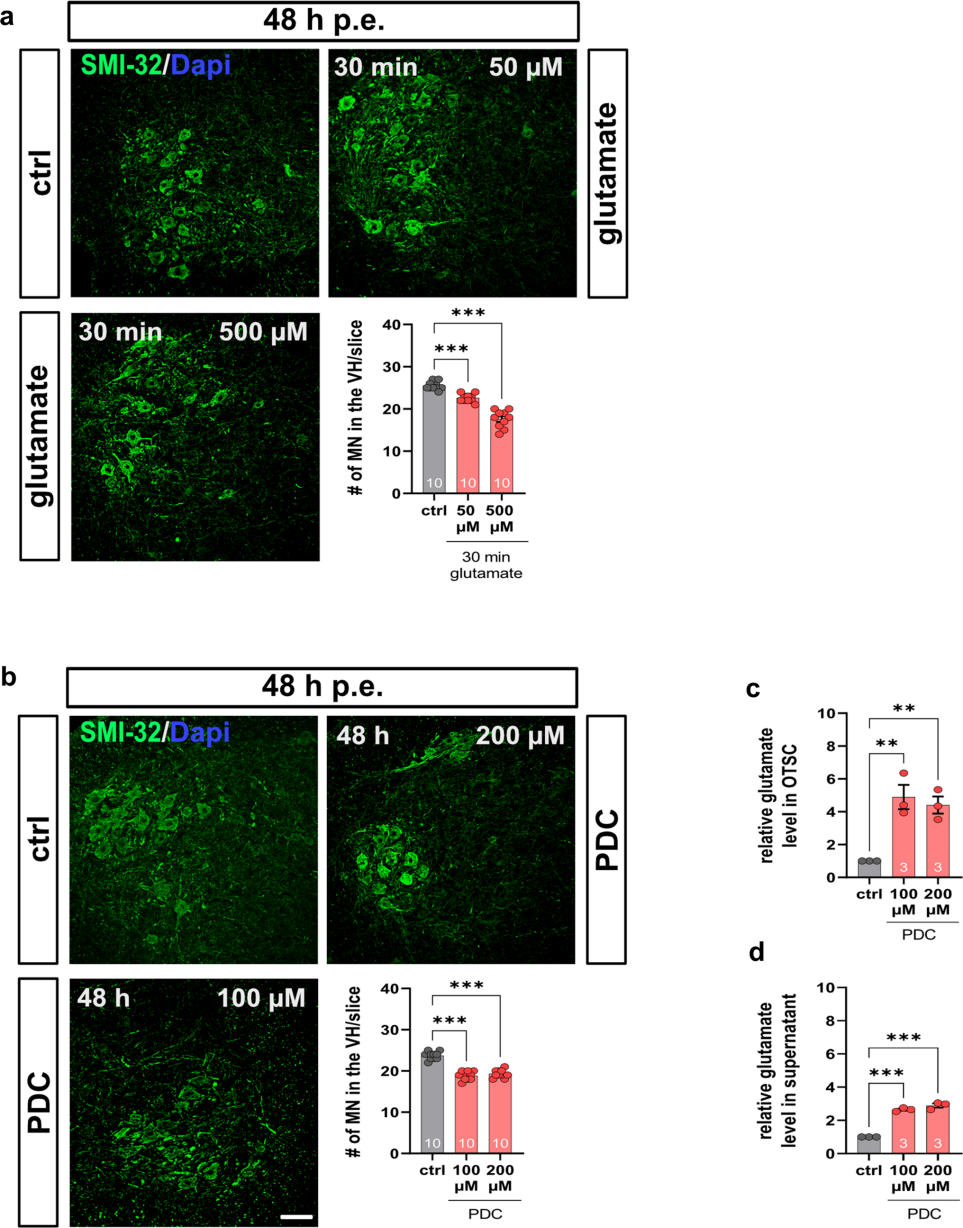
Ex vivo model of glutamate-mediated MN death. **a** Immunostaining of spinal MNs (SMI-32, green) in OTSCs from wt mice 48 h post-exposure (p.e.) to a 30 min pulse of 50 or 500 µM glutamate. Nucleic DNA was stained with Dapi (blue). Forty-eight hours after glutamate pulse, the number of spinal MNs was reduced (*p* < 0.001). **b** Immunostaining of spinal MNs (SMI-32, green) in OTSC from wt mice after exposure to the EAAT1 inhibitor PDC for 48 h. The inhibition of EAAT1 reduced spinal MNs after 48 h (*P* < 0.001). **c** PDC treatment enhanced glutamate level in OTSC (*p* > 0.01) **d** PDC treatment enhanced glutamate level in supernatant of OTSCs (*p* > 0.001) *n* = 10 slices per condition. Scale bar: 20 µm. Abbreviations: EAAT1, excitatory amino acid transporter 1; MN, motor neuron; OTSC, organotypic spinal cord slice cultures, PDC, L-*trans*-pyrrolidine-2,4-dicarboxylic acid; SMI-23, neurofilament heavy polypeptide; wt, wild type; #, number per slice. *P* values: *p < 0.05, **p < 0.01, or ***p < 0.001

The influence of glutamate on spinal MN survival was determined using spinal cord slice cultures prepared from wt mice. Forty-eight hours after glutamate exposure of either 50 or 500 µM for 30 min, the number of spinal MNs in spinal cord slices was reduced (*p* < 0.0001) (Fig. 5a). Exposing the spinal cord slice cultures to PDC (100 or 200 µM) for 48 h reduced the number of spinal MNs (*p* < 0.0001) (Fig. 5b). The uptake of glutamate in PDC treated cultures was reduced, resulting in increased glutamate levels in the tissue (100 µM PDC: *p* = 0.006; 200 µM PDC: *p* = 0.007) and supernatant (*p* < 0.0001), suggesting impaired glutamate uptake as driving force for the degeneration of spinal MNs (Fig. 5c, d).

### Upregulation of EAAT1 in SMA mice prevented motor neuron loss

To evaluate the importance of EAAT1 dysfunction to late-onset SMA pathogenesis, SMA and wt mice were treated with AA, a lipid known to inhibit astrocyte activation and increase the expression of the EAAT1 protein [36]. AA was repetitively administered or a vehicle to SMA mice by i.p. injections.

**Fig. 6:**
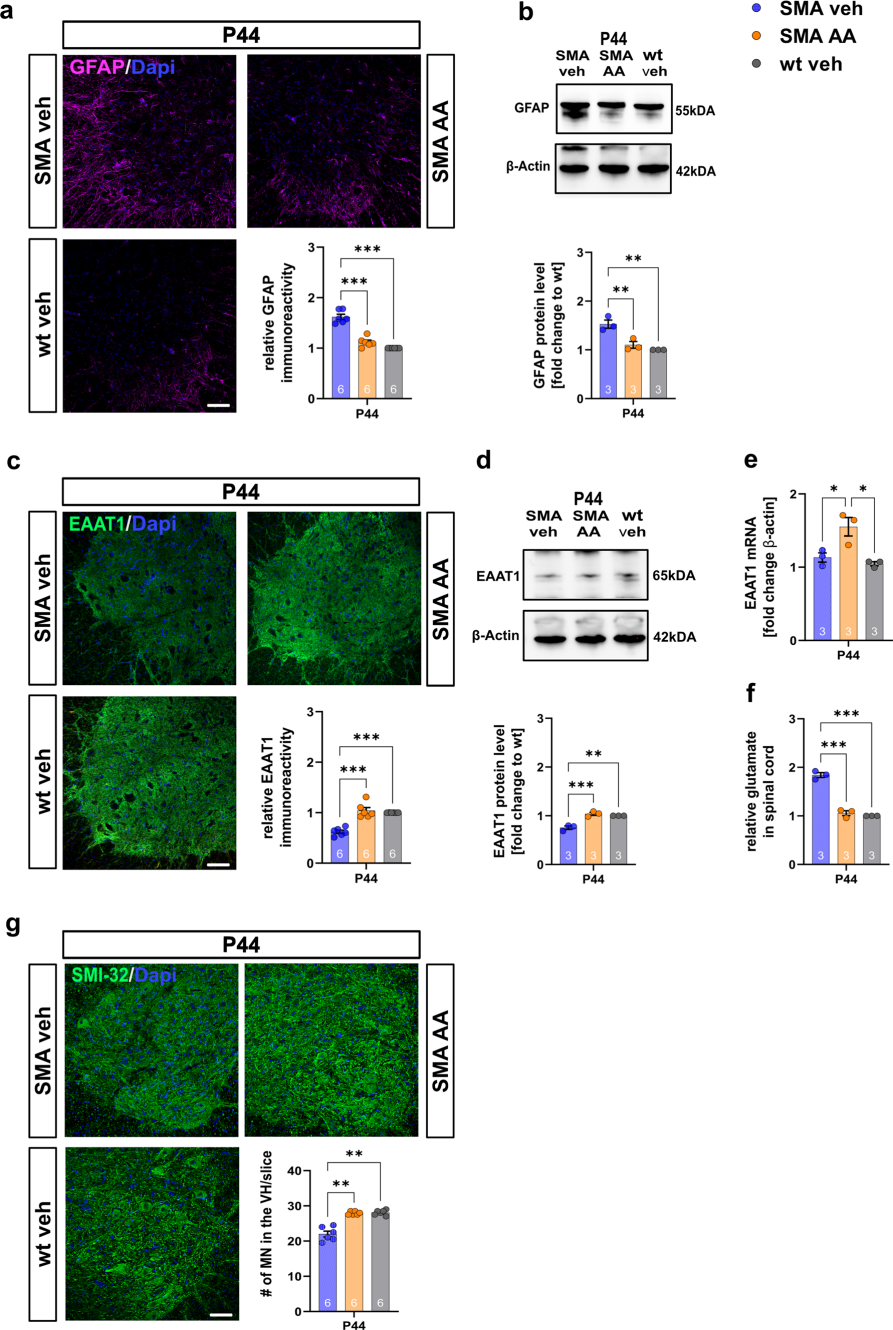
In vivo administration of AA inhibits astrocyte activation, enhances EAAT1 expression, and avoids MN loss. **a** Immunostaining of GFAP (magenta) in the spinal cord ventral horns of wt or SMA mice treated with vehicle or AA at P44. Nucleic DNA was stained with Dapi (blue). SMA mice treated with vehicle showed elevated protein levels of GFAP compared to wt mice (*p* < 0.001). When SMA mice were treated with AA, GFAP protein levels were reduced compared to vehicle-treated SMA animals (*p* < 0.001). **b** Western blot analysis of the GFAP protein level in the spinal of wt or SMA mice treated with vehicle or AA at P44. Similar effects as described for immunostaining were observed (*p* < 0.01). **c** Immunostaining of EAAT1 (green) in the spinal cord ventral horns of wt or SMA mice treated with vehicle or AA at P44. Nucleic DNA was stained with Dapi (blue). SMA mice treated with vehicle showed reduced protein levels of EAAT1 compared to wt mice (*p* < 0.001). When SMA mice were treated with AA, EAAT1 protein levels were elevated compared to vehicle-treated SMA animals (*p* < 0.001). **d** Western blot analysis of EAAT1 protein level in the spinal cord tissue of wt or SMA mice treated with vehicle or AA at P44. Similar effects as described for immunostaining were observed (*p* < 0.01 to *p* < 0.001). **e** EAAT1 mRNA level was increased in AA-treated SMA mice (*p* < 0.05). In contrast, the mRNA level was not affected in vehicle-treated SMA mice (*p* > 0.05) **f** Measurement of glutamate levels in the spinal cord tissue of wt or SMA mice treated with vehicle or AA using a glutamate assay kit. The glutamate level in the spinal cord tissue of vehicle-treated SMA mice was elevated compared to wt mice (*p* < 0.001). When SMA mice were treated with AA, the glutamate level in their spinal cord tissue was reduced to the control level (*p* < 0.001). **g** Immunostaining of spinal MN (SMI-32, green) in the spinal cord ventral horns of wt or SMA mice treated with vehicle or AA at P44. Nucleic DNA was stained with Dapi (blue). Vehicle-treated SMA mice showed a reduced number of MNs compared to wt mice at P44 (*p* < 0.01). When SMA mice were treated with AA, the number of MNs stayed at the level of wt mice (*p* < 0.01 to vehicle SMA; *p* > 0.05 to wt). *n* = 6 animals per condition for immunostaining. Three slices per lumbar spinal cord were investigated. Each data point reflects the mean of three spinal cord slices per animal. *N* = 3 animals per condition for Western blot analysis, qPCR analysis, and glutamate measurements. Scale bar: 20 µm. Abbreviations: AA, arundic acid; EAAT1, excitatory amino acid transporter 1; GFAP, glial fibrillary acid protein; MN, motor neuron; mRNA, messenger ribonucleic acid; P, postnatal day; qPCR, real-time polymerase chain reaction; SMA, spinal muscular atrophy; SMI-23, neurofilament heavy polypeptide; wt, wild type; #, number per slice. *P* values: *p < 0.05, **p < 0.01, or ***p < 0.001

Administration of AA at P28, when the EAAT1 was reduced but prior to MN loss, prevented astrocytic activation (as measured by GFAP) in SMA mice at P44 (*p* < 0.0001, Fig. 6a; SMA veh vs wt veh: *p* = 0.006; SMA veh vs SMA AA: *p* = 0.003, Fig. 6b) (Supplementary Fig. 4, online resource).

In SMA mice treated with AA, EAAT1 protein and mRNA levels were upregulated compared to vehicle-treated SMA mice (*p* < 0.0001, Fig. 6c; *p* < 0.001, Fig. 6d; *p* = 0.023, Fig. 6e) (Supplementary Fig. 5, online resource) with a reduction of glutamate levels (*p* < 0.0001) (Fig. 6f), while the loss of MNs was prevented in SMA mice (*p* = 0.001) (Fig. 6g. Supplementary Fig. 8, online resource).

**Fig. 7:**
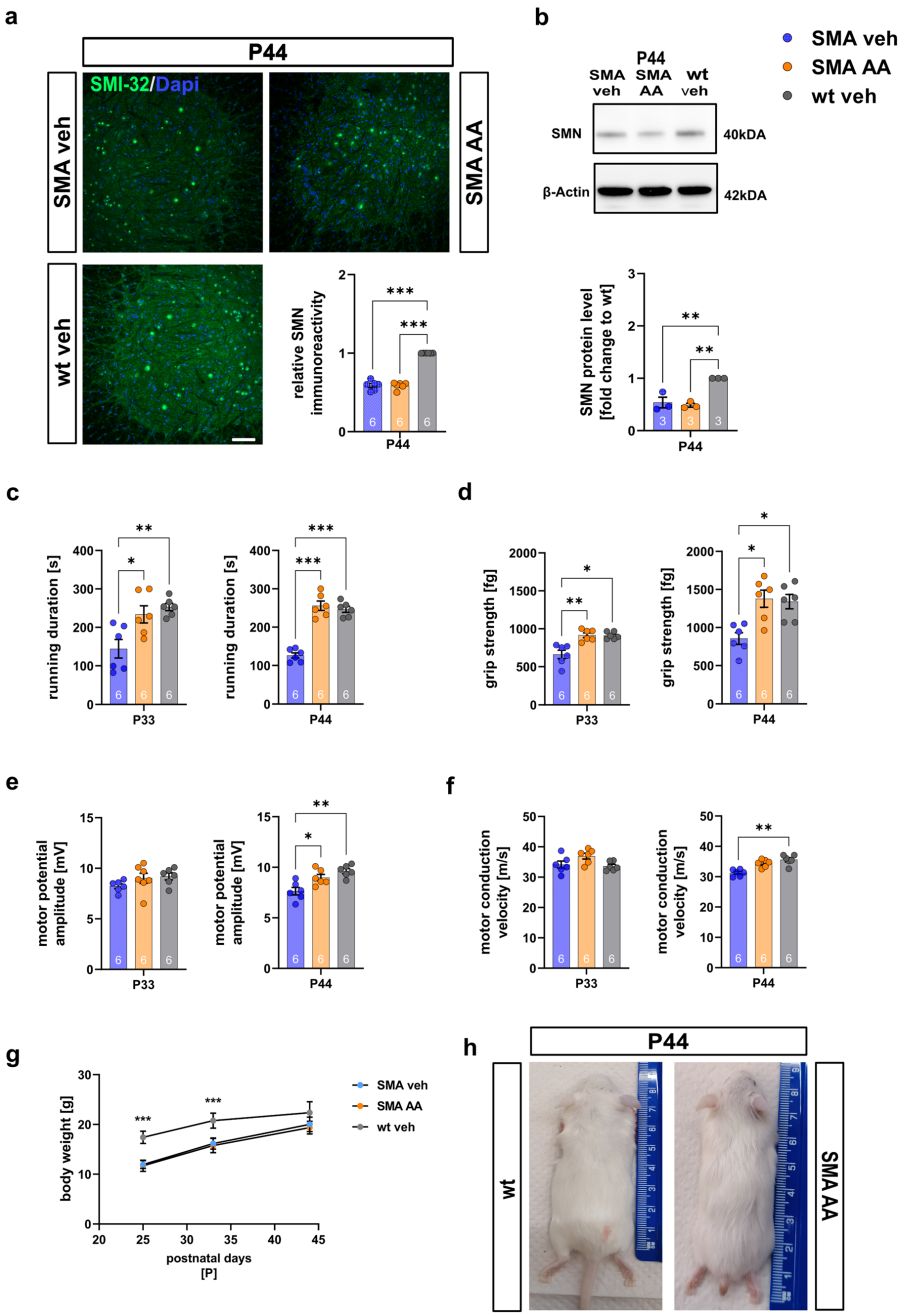
Arundic acid preserves motor functions and electrophysiological properties of late-onset SMA mice in an SMN-independent manner. **a** Immunostaining of SMN (green) in the spinal cord ventral horns of wt or SMA mice treated with vehicle or AA at P44. Nucleic DNA was stained with Dapi (blue). The relative SMN protein level in the ventral horns of SMA mice treated with vehicle or AA was reduced compared to wt vehicle mice (*p* < 0.001), confirming that the effects of AA were SMN-independent. **b** Western blot analysis of SMN protein levels in spinal cord tissue of vehicle-treated wt, or vehicle- or AA-treated SMA mice at P44. Beta-actin was used as a loading control. SMA mice showed reduced SMN protein levels (*p* < 0.01) compared to wt mice. **c** Rotarod assessment of wt or SMA mice treated with vehicle or AA at P33 and P44. Vehicle-treated SMA mice showed a reduced running duration on the rotarod compared to wt mice at P33 (*p* < 0.01) and P44 (*p* < 0.001). When SMA mice were treated with AA, the running duration was enhanced compared to the vehicle-treated SMA mice at P33 (*p* < 0.05) and P44 (*p* < 0.001). **d** Grip strength measurements of wt or SMA mice treated with vehicle or AA at P33 and P44. Vehicle-treated SMA mice showed reduced grip strength at P33 (*p* < 0.05) and P44 (*p* < 0.05) compared to wt mice. The grip strength of AA-treated SMA mice was enhanced at P33 (*p* < 0.01) and P44 (*p* < 0.05) compared to vehicle-treated SMA mice. **e** MPA measurements of wt or SMA mice treated with vehicle or AA at P33 and P44. At P44, MPA was reduced in vehicle-treated SMA mice compared to AA-treated SMA (*p* < 0.05) and vehicle-treated wt mice (*p* < 0.01). **f** At P44, motor conduction velocity was slightly reduced in vehicle-treated SMA mice compared to wt mice (*p* < 0.01). **g** The body weight of SMA mice was not altered by AA. **h** The phenotype of SMA mice was not affected by AA treatment. *N* = 6 animals per condition for immunostaining. Three slices per lumbar spinal cord were investigated. Each data point reflects the mean of three spinal cord slices per animal. *N* = 3 animals per condition for Western blot analysis and glutamate measurements. *N* = 6 animals per condition for motor behavior and electrophysiological experiments. Scale bar: 20 µm. *AA* arundic acid, *MPA* motor potential amplitude, *P* postnatal day, *SMA* spinal muscular atrophy, *SMN*,survival of motor neuron, *wt*,wild type. *P* values: *p < 0.05, **p < 0.01, or ***p < 0.001

A direct impact of AA on the SMN level was not detected (*p* > 0.999) compared to SMA mice treated with vehicle, suggesting these effects were SMN-independent (Fig. 7a and b. Supplementary Fig. 4, online resource).

### Motor functions and electrophysiological properties in SMA mice were preserved by AA

Treating SMA mice with AA increased the running duration on the rotarod compared to vehicle-treated SMA mice at P33 (*p* = 0.012) and P44 (*p* < 0.0001). Vehicle-treated SMA mice showed a reduced running duration on the rotarod compared to wt mice at P33 (*p* = 0.005) and P44 (*p* < 0.0001) (Fig. 7c).

Grip strength was increased in AA-treated SMA mice compared to vehicle-treated SMA mice at P33 (*p* = 0.007) and P44 (*p* = 0.012). In contrast, vehicle-treated SMA mice had reduced grip strength compared to wt mice (P33: *p* = 0.012; P44:* p* = 0.024) (Fig. 7d).

The motor potential amplitude (MPA) in AA-treated SMA mice was increased compared to vehicle-treated SMA mice at P44 (*p* = 0.015), while vehicle-treated SMA mice showed reduced MPA compared to wt mice (*p* = 0.01). No difference between AA-treated SMA and vehicle-treated wt mice was observed at P33 (*p* > 0.335) (Fig. 7e).

At P33, no change in motor conduction velocity (MCV) was measured between the compared groups (*p* > 0.55), while MCV was slightly reduced in vehicle-treated SMA mice at P44 (*p* = 0.002) (Fig. 7f).

AA did not influence body weight or tail necrosis progression (Fig. 7g, h).

### The number of muscle fibers and the structures of the NMJ in SMA mice were preserved by AA

**Fig. 8:**
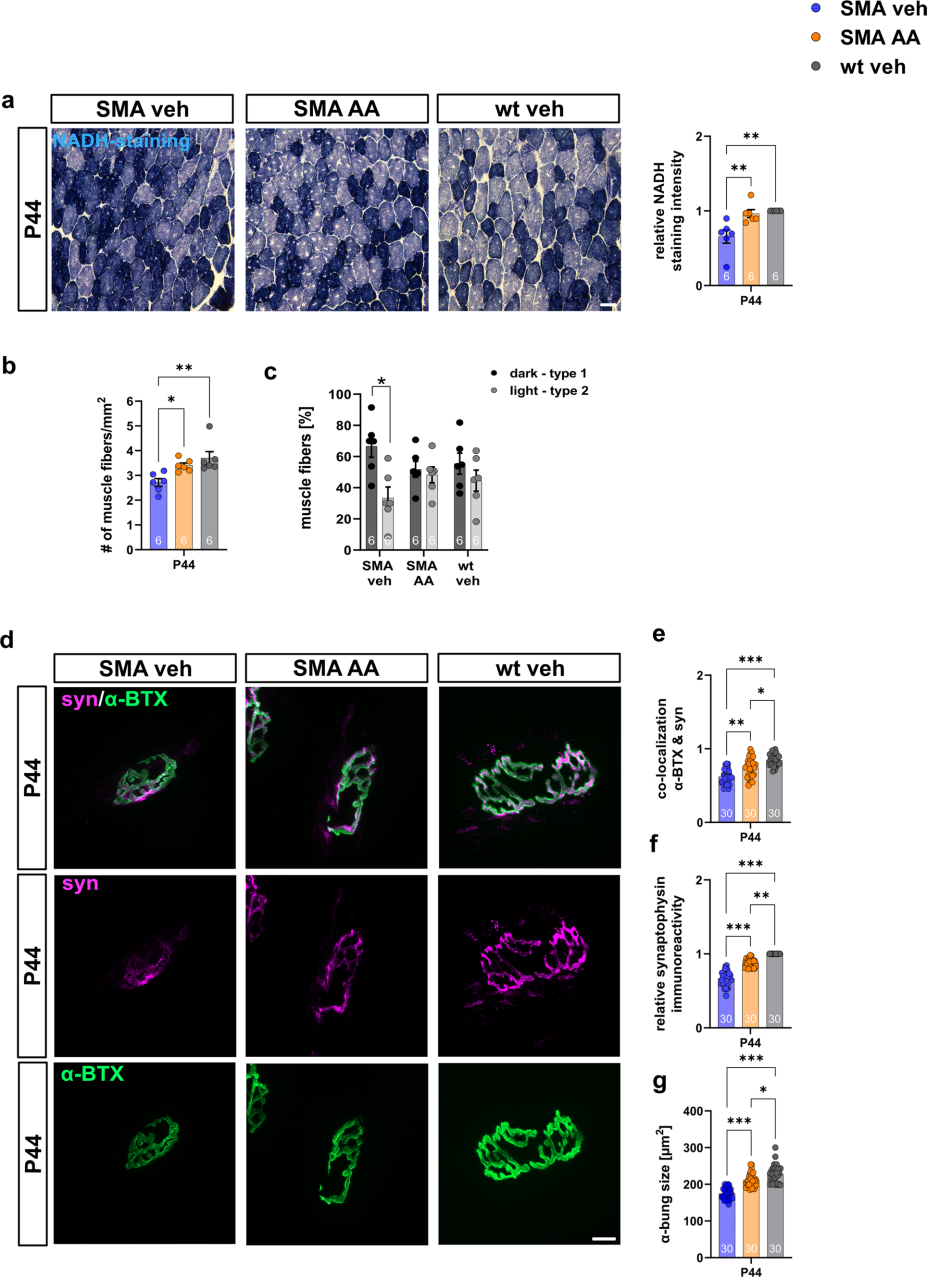
Arundic Acid preserves NMJ and loss of type 2 muscle fibers in the M. *tibialis anterior*. **a** NADH staining of M. *tibialis anterior* of late-onset SMA mice treated with AA or vehicle compared to wt mice at P44. Type 1 muscle fibers are stained dark, while type 2 fibers are stained light. NADH staining intensity was reduced in vehicle-treated SMA mice (getting darker) compared to wt (*p* < 0.01) and AA-treated SMA mice (*p* < 0.01). **b** Total number of muscle fibers was reduced in vehicle-treated SMA mice (*p* < 0.01) but not in AA-treated SMA mice (*p* > 0.05 or *p* < 0.05). **c** Number of light-stained type 2 fibers were reduced in vehicle-treated SMA mice (*p* < 0.05), while SMA mice treated with AA were not affected (*p* > 0.05). **d** Immunostaining of presynaptic synaptophysin (magenta) and postsynaptic α-BTX (green) at the NMJ of late-onset SMA mice treated with AA or vehicle compared to wt mice at P44. **e** In vehicle-treated SMA mice co-localization of synaptic proteins was reduced (*p* < 0.001). In AA-treated SMA mice co-localization was enhanced compared to vehicle-treated SMA mice (*p* < 0.01) but was reduced to wt mice (*p* < 0.05). **f** Synaptophysin immunoreactivity was reduced in vehicle-treated SMA mice (*p* < 0.001), but enhanced in SMA mice treated with AA compared to vehicle-treated SMA mice (*p* < 0.001). Synaptophysin immunoreactivity was still reduced compared to wt mice (*p* < 0.01). **g** Presynaptic size of the NMJ was reduced in vehicle-treated SMA (*p* < 0.001) but enhanced in AA-treated SMA mice (*p* < 0.001). The presynaptic size in AA-treated SMA mice was slightly reduced to wt mice (*p* < 0.05). *n* = 6 animals per condition for NADH staining. Three slices per animal were investigated. *N* = 30 neuromuscular endplates were analyzed in total per condition. Five endplates per animal, 6 animals per condition. Scale bar: 50 µm. *AA* arundic acid, *α-BTX* α-bungarotoxin, *NADH* nicotinamide adenine dinucleotide hydrogen, *NMJ* neuromuscular junction, *SMA* spinal muscular atrophy, *wt*,wild type. *P* values: *p < 0.05, **p < 0.01, or ***p < 0.001

For analysis of muscle fiber numbers after AA treatment, NADH staining of M. *tibialis anterior* slices was performed at P44. When SMA mice were treated with AA, staining intensity was enhanced compared to vehicle-treated SMA mice (*p* = 0.005; *p* = 0.003). No difference to wt mice was observed (*p* = 0.681) (Fig. 8a). While in vehicle-treated SMA mice the number of muscle fibers was reduced (*p* = 0.006), in AA-treated SMA mice muscle fibers were not affected (*p* = 0.272) (Fig. 8b). In SMA mice treated with vehicle the number of light-stained type 2 fibers was reduced (*p* = 0.042). In comparison, in AA-treated SMA mice the distribution of fiber types was similar to wt mice (*p* = 0.968) (Fig. 8c).

The potential protective effect of AA on the NMJ was examined by immunostaining of synaptophysin as presynaptic and α-BTX as a postsynaptic marker at P44 (Fig. 8d). In AA-treated SMA mice the colocalization of pre-and postsynaptic markers was increased compared to vehicle-treated SMA mice (*p* = 0.005) but slightly reduced to wt mice (*p* = 0.032) (Fig. 8e). Synaptophysin immunoreactivity and postsynaptic area were reduced in vehicle-treated SMA mice (*p* < 0.0001, Fig. 8f; *p* < 0.0001, Fig. 8e). When SMA mice were treated with AA, synaptophysin and the postsynaptic area were still slightly reduced to wt mice (*p* = 0.006, Fig. 8f; *p* = 0.032, Fig. 8e) but enhanced compared to vehicle-treated SMA mice (*p* < 0.0001) (Fig. 8f, g).

### Impaired glutamate homeostasis in late-onset SMA patients

**Fig. 9:**
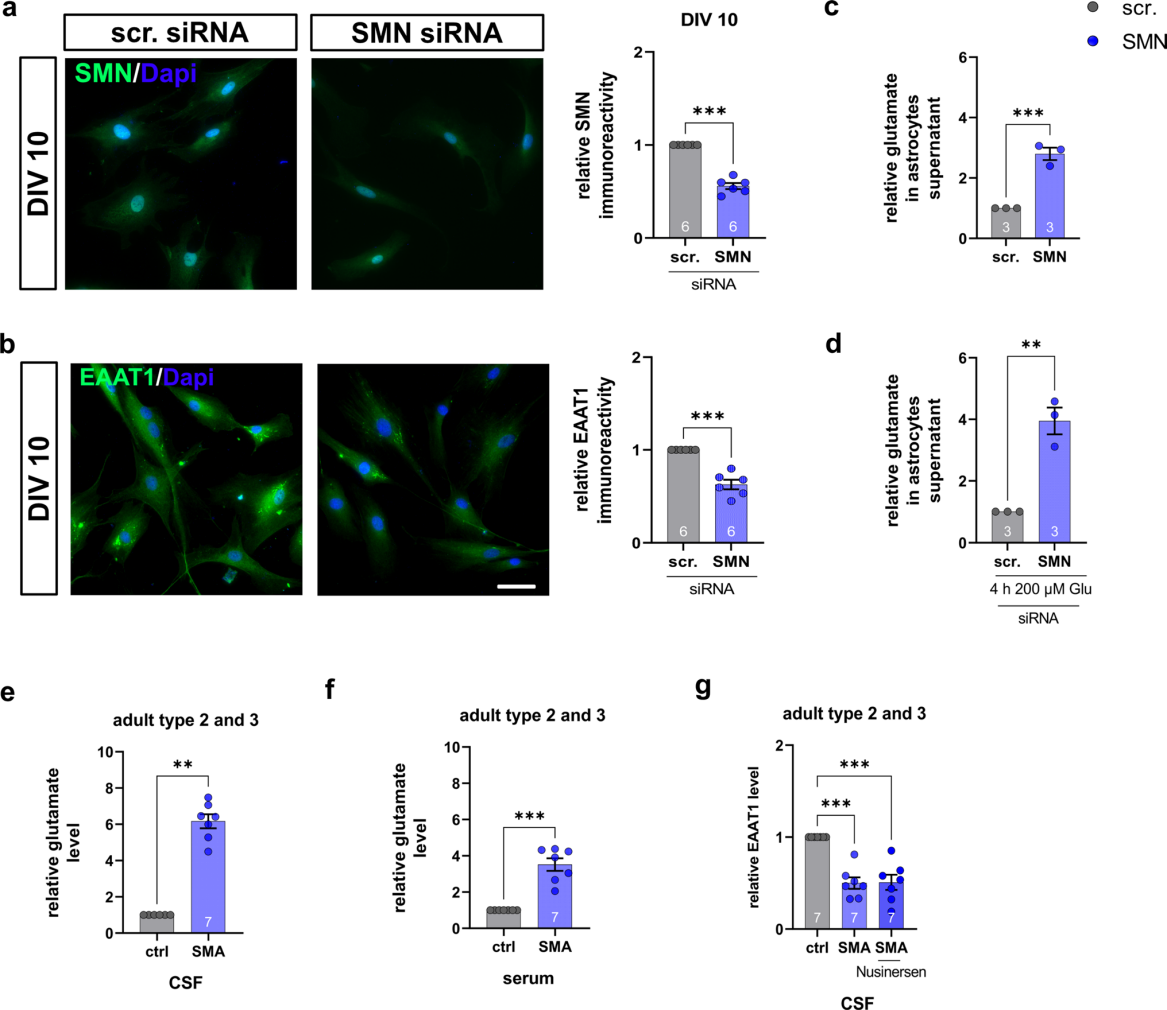
Impaired glutamate homeostasis in induced human astrocytes and human CSF and serum samples. **a** Immunostaining of SMN (green) in induced human astrocytes transfected with scrambled or *SMN* siRNA at DIV 10. Nucleic DNA was stained with Dapi (blue). Astrocytes transfected with SMN siRNA showed reduced SMN protein levels (*p* < 0.001). **b** Immunostaining of EAAT1 (green) in induced human astrocytes transfected with scrambled or SMN siRNA at DIV 10. Nucleic DNA was stained with Dapi (blue). Astrocytes transfected with SMN siRNA showed reduced EAAT1 protein levels compared to scrambled siRNA-transfected cells (*p* < 0.001). **c** Induced human astrocytes transfected with SMN siRNA but not exposed to glutamate showed an increased release of glutamate compared to astrocytes transfected with scrambled siRNA (*p* < 0.001). **d** When scrambled or SMN siRNA-transfected induced human astrocytes were exposed to 200 µM glutamate for 4 h, SMN-deficient induced human astrocytes showed reduced uptake of glutamate (*p* < 0.01). **e** CSF samples of SMA type 2 and 3 patients showed elevated glutamate levels compared to controls (*p* < 0.01). **f** Serum samples of SMA type 2 and 3 patients showed elevated glutamate levels compared to controls (*p* < 0.001*).* **g** In CSF samples of SMA type 2 and type 3 patients EAAT1 level was reduced prior to treatment. Nusinersen-treatment did not affect EAAT1 level. *n* = 6 independent experiments for immunostaining. For each experiment, > 50 cells were analyzed per condition. *n* = 3 independent experiments for functional assays. *n* = 7 patients for glutamate assay of CSF or serum samples. Scale bar: 50 µm. *DIV* days in vitro; EAAT1, excitatory amino acid transporter 1, *siRNA* small interfering ribonucleic acid, *SMA* spinal muscular atrophy, *SMN* survival of motor neuron. *P* values: *p < 0.05, **p < 0.01, or ***p < 0.001

To provide translational evidence for the role of EAAT1-driven glutamate toxicity in SMA, we investigated induced human astrocytes from healthy individuals, CSF, and serum samples from late-onset SMA patients. In astrocytes converted from human fibroblasts transfected with *SMN*1 siRNA, SMN and EAAT1 levels were reduced (*p* = 0.0001) (Fig. 9a, b). In addition, glutamate measured in the supernatant of SMN-deficient induced human astrocytes was increased (*p* < 0.0001) (Fig. 9c). After exposure to 200 µM glutamate for 4 h, SMA-like induced human astrocytes showed reduced uptake of glutamate (*p* = 0.0056) (Fig. 9d).

Glutamate levels in human CSF and serum samples of late-onset SMA type 2 and 3 patients were elevated compared to healthy individuals (*p* = 0.01, Fig. 9e; *p* = 0.001, Fig. 9f). Furthermore, EAAT1 levels in CSF samples of SMA type 2 and 3 patients were reduced (*p* < 0.0001). In nusinersen treated patients, no change in EAAT1 CSF-level compared to the SMA baseline conditions was detectable (*p* = 0.996), suggesting EAAT1 to be an SMN-independent target (Fig. 9g). EAAT1 protein was not detectable in serum samples of SMA patients or healthy individuals (data not shown).

## Discussion

Our study focused on the role of spinal cord astrocytes in the pathogenesis of late-onset SMA. We provide evidence for the crucial involvement of glutamate uptake protein EAAT1 by elevating the glutamate levels in the spinal cord using a translational approach. Furthermore, we provide a neuroprotective strategy, enhancing EAAT1 expression by administrating astrocyte-modulating lipid AA in vivo, qualifying EAAT1 as a potential therapeutic target for the SMN-independent treatment of SMA.

The SMA mouse model reflects a less severe disease course of SMA [41]. These mice show reduced expression of SMN protein in the spinal cord tissue, a normal lifespan, and signs of mild hindlimb paresis due to MN loss, loss of type 2 muscle fibers, and denervated NMJ as well as reduced body weight, as in other SMA models [30, 57]. In severe SMA mouse models, metabolic issues and changes in body weight could be reversed by different used diets; either a low or high-fat diet can lead to increased body weight and the span of survival [10, 15] and are a topic of current discussions. In contrast to severe SMA models, the late-onset model develops necrotic ear and tail tissue during the disease course caused by tissue ischemia or skeletal muscle denervation, combined with an enhanced life-span of this model [32, 54, 58, 67]. Those necrotic processes do not belong to the usual human SMA phenotype.

Recent studies have indicated that SMA is no longer an exclusive motor neuron disorder due to the wide expression of the SMN protein in the CNS, suggesting SMA is a multisystem disorder [71]. The crucial contribution of astrocytes to diseases of the CNS has been demonstrated for disorders, such as Alzheimer’s disease or epilepsy [6, 31, 53, 56]. Astrocytes are also suggested to be critically involved in the pathogenesis of SMA. Most of these studies demonstrated early enhanced expression of GFAP as a sign of increased reactivity of spinal astrocytes before the loss of spinal MN appeared in severe mouse models or have recently demonstrated higher levels in CSF of SMA patients [22, 33, 45, 59].

However, none of these studies has confirmed the contribution of astrocytes to MN loss in a functional-mechanistic and translational approach resulting in the identification of astrocytic proteins as potential new therapeutic targets.

A major function of astrocytes is regulating extracellular glutamate levels by taking up excitatory neurotransmitters from the synaptic cleft via EAAT proteins, thus protecting neurons from excitotoxicity [29, 60, 61]. Dysfunction in astrocyte-mediated glutamate transport has been identified in CNS diseases, such as epilepsy, Alzheimer’s disease, and amyotrophic lateral sclerosis (ALS) [21, 29, 64, 65]. Here, we provide evidence for EAAT1-mediated glutamate toxicity as a significant driving force for MN degeneration in late-onset SMA.

The early observed EAAT1 reduction suggested a glutamate-mediated MN loss, which we confirmed by elevated glutamate levels detected in the spinal cord and a similar pattern in SMA-like astrocytes. However, this effect was only on protein but not on mRNA level, suggesting post-transcriptional modulation of EAAT1 mRNA by processes, such as methylation or micro-(mi) RNA involvement [5, 37]. Due to our cell culture experiments on mouse and human astrocytes, we can assume an autonomous reduction of EAAT1 in spinal astrocytes as a result of SMN protein lack rather than a response to the exogenous impact of other cell types, such as microglia or different immune cells. Furthermore, the direct relation between EAAT1 dysfunction and disturbed glutamate uptake was confirmed using PDC in our ex vivo model.

The increased glutamate levels in supernatants from SMN-deficient mice or induced human astrocytes in vitro models suggest that altered astrocytic glutamate release may be an additional source for glutamate enhancement, besides neuronal release, in the spinal cord tissue. These results are complemented by elevated glutamate levels in the CSF and serum samples of SMA type 2 and 3 patients. In addition, the EAAT1 level was reduced in CSF samples of SMN patients at baseline conditions, proving its clinical relevance. When SMA patients were treated with nusinersen, EAAT1 level in the CSF was not changed compared to baseline conditions, suggesting EAAT1 to be an SMN-independent potential therapeutic target.

Excitotoxicity by glutamate as a cause of neuronal degeneration has been widely accepted for other disorders, such as ALS [17, 34]. Glutamate acts as a critical messenger for the correct physiological function of MNs and astrocytes and their interactions [20, 44, 63]. Elevated glutamate levels are toxic for MNs and their surrounding cells [17, 48]. Inadequate or perturbed glutamate uptake from astrocytes can increase hyperexcitation of N-methyl-D-aspartate and α-amino-3-hydroxy-5-methyl-4-isoxazole propionic acid (AMPA) receptors. Motor neurons seem particularly sensitive to AMPA-mediated glutamate toxicity, increasing intracellular calcium levels due to calcium-permeable AMPA receptor subunits, promoting MN death [11, 38, 68]. Here, we demonstrate the involvement of EAAT1 dysfunction in this process using in vitro and in vivo experiments. The contribution of AMPA or other glutamate receptors to SMA pathogenesis needs to be addressed in further studies.

Arundic acid is a lipid shown to inhibit astrocytic activity, enhance the expression of EAAT1 in the brain, and increase astrocytic glutamate uptake [36, 70]. In a study on intracerebral hemorrhage, a severe stroke subtype, the direct administration of AA into the left striatum of mice enhanced grip strength and walking test outcome [13]. In our study, administering AA to SMA mice, when EAAT1 expression was reduced but prior to the beginning of MN loss, prevented this loss by enhancing the EAAT1 expression and reducing the glutamate level within SMA mice. The enhanced expression of EAAT1 mRNA, suggests a direct transcriptional effect of AA on EAAT1. AA induces nuclear translocation of NF-κB and its binding to the EAAT1 promotor. In addition, the activation of AKT and ERK signaling pathways by AA as an EAAT1-enhancing mechanism is discussed [36]. These results confirmed the hypothesis of EAAT1-mediated glutamate toxicity as a crucial mechanism in the early pathogenesis of late-onset SMA. Besides cellular changes, AA administration also ameliorated the phenotypic disease manifestations in our SMA mouse model. AA prevented the loss of type 2 muscle fibers in late-onset SMA mice, assuming a direct impact of AA on muscle tissue.

Interestingly, AA slowed down the denervating process of the neuromuscular junctions significantly but did not fully protect them. Such a protective effect could be explained by the prevented loss of spinal MN after enhancing EAAT 1 protein expression or by an additional beneficial effect of AA on Schwann cells. These cells are crucial for structuring the NMJ during their development or regeneration by guiding motor axons [3]. Therefore, an earlier administration timepoint should be suggested to fully protect neuromuscular junctions by AA. However, the direct effect of AA on Schwann cells needs to be addressed in future studies.

The repetitive in vivo administration of AA to SMA mice did not affect the SMN protein level, identifying its therapeutic effects to be SMN-independent. In addition, further SMN-independent targets, such as Rho kinase (ROCK), extracellular regulated kinase (ERK), and the c-Jun N-terminal Kinase (JNK) were also identified in different studies [7–9, 23, 25–28, 55, 62].

Current SMN-enhancing drugs have an impressive benefit in SMA patients. Nevertheless, not all motor functionalities can be increased in patients using these drugs [24, 52]. In addition, delayed administration, especially to late-onset SMA patients, negatively impacts the efficacy of the drug due to the irreversible loss of spinal MN. Therefore, complementary strategies, such as enhancing EAAT1 expression in spinal astrocytes, could have synergistic therapeutic potential.

Our study demonstrates the critical involvement of spinal astrocytes in the pathogenesis of late-onset SMA in a functional and translational approach. For the first time, glutamate toxicity mediated by the downregulation of EAAT1 has been identified as a significant driving force for early MN loss in late-onset SMA, and qualifies EAAT1 as a potential therapeutic target for SMN-independent treatment strategies to complement SMN-enhancing drugs in late-onset SMA therapy.

## Supplementary Information

Below is the link to the electronic supplementary material.

Supplementary file1 (DOCX 8207 KB)

## Data Availability

The data that support the study’s findings are available from the corresponding author (L-IS) upon reasonable request.

## Declarations

### Research involving human and/or animal participants

All experiments were conducted under the animal welfare guidelines of the University of Duisburg-Essen. Furthermore, the SMA mouse model used and in vivo experiments were approved by the State Agency for Nature, Environment and Consumer Protection (LANUV) in North Rhine-Westphalia, Germany (reference number 81–02.04–2020.A335; 81–02.03.2021.A078).
