# Supplementary material for: Spinal astrocyte dysfunction drives motor neuron loss in late-onset spinal muscular atrophy
Source: Acta Neuropathol. 2023 Mar 17;145(5):611–35. doi: 10.1007/s00401-023-02554-4 (PMC10119066; doi:10.1007/s00401-023-02554-4)
Supplement: Supplementary file 1 — Supplementary file1 (DOCX 8207 KB) [file 401_2023_2554_MOESM1_ESM.docx]

Spinal astrocyte dysfunction drives motor neuron loss in late-onset spinal muscular atrophy

Linda-Isabell Schmitt^1^, Christina David^1^, Rebecca Steffen^1^, Stefanie Hezel^1^, Andreas Roos^2^, Ulrike Schara-Schmidt^2^, Christoph Kleinschnitz^1^, Markus Leo^1^ and Tim Hagenacker^1^

^1^ Department of Neurology, Center for Translational Neuro- and Behavioral Sciences (C-TNBS), University Hospital Essen, Hufelandstr. 55, 45147 Essen, Germany

^2^ Department of Pediatrics 1, Division of Neuropediatrics, Center for Translational Neuro- and Behavioral Sciences (C-TNBS), University Hospital Essen, Hufelandstr. 55, 45147 Essen, Germany

***corresponding author**

Linda-Isabell Schmitt

Department of Neurology

Center for Translational Neuro- and Behavioral Sciences (C-TNBS)

University Hospital Essen

Hufelandstr. 55

45147 Essen, Germany

[Linda-Isabell.Schmitt@UK-Essen.de](mailto:Linda-Isabell.Schmitt@UK-Essen.de)

**Supplementary material 1**

Western blot of wild-type and late-onset SMA mice (3 individual mice per condition). Total protein, SMN (anti-rabbit Novus Biologicals) and Actin (anti-rabbit Abcam) staining; red box indicates used WB in the manuscript.


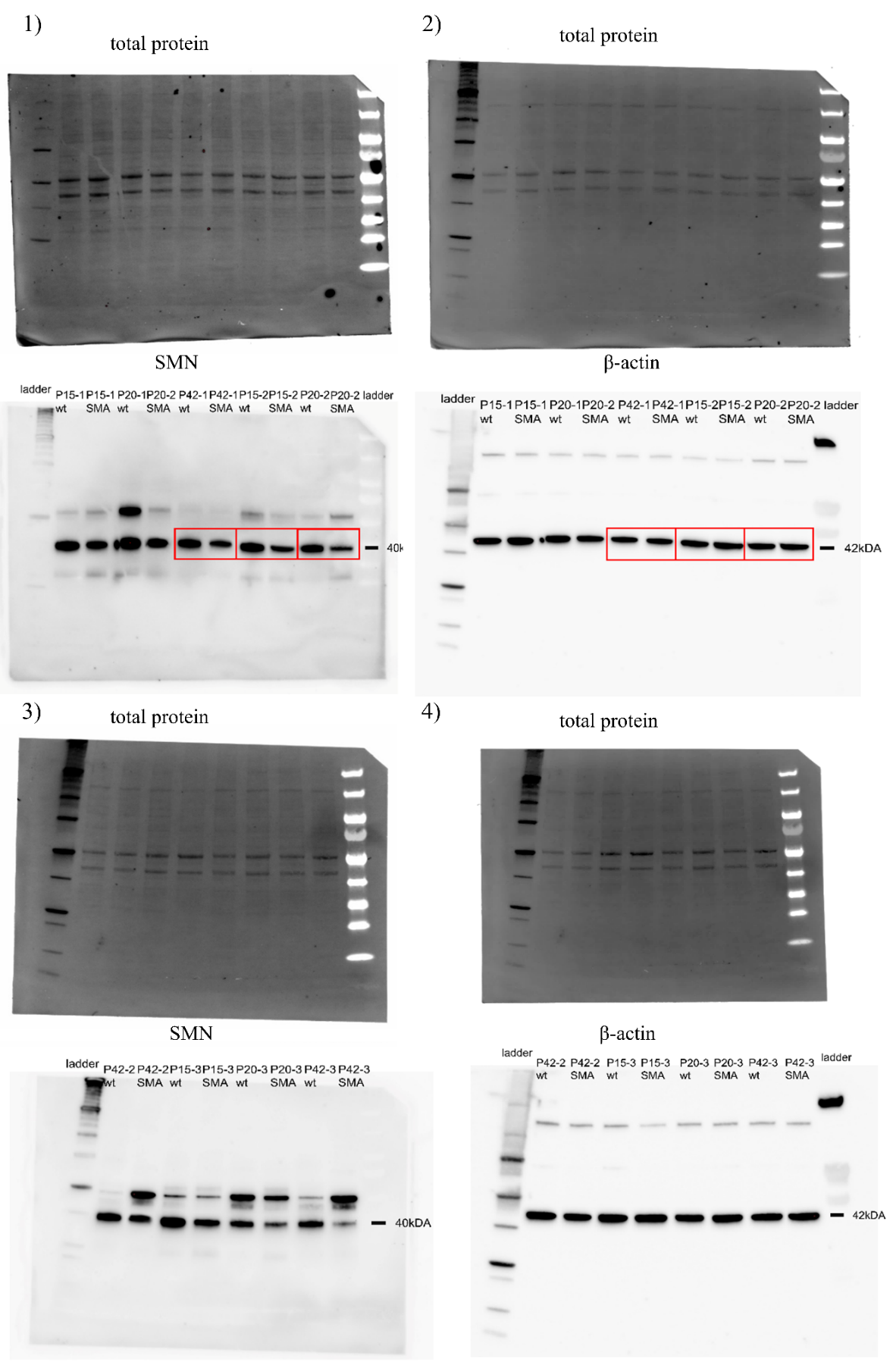


**Supplementary Figure 1:** Total protein staining of the membrane. Western Blot of SMN and Actin of wild-type and late-onset SMA mice at P15, P20 and P42.

Western blot of wild-type and late-onset SMA mice (3 individual mice per condition). Total protein, GFAP (anti-mouse Sigma Aldrich) and Actin (anti-rabbit Abcam staining; red box indicates used WB in the manuscript.


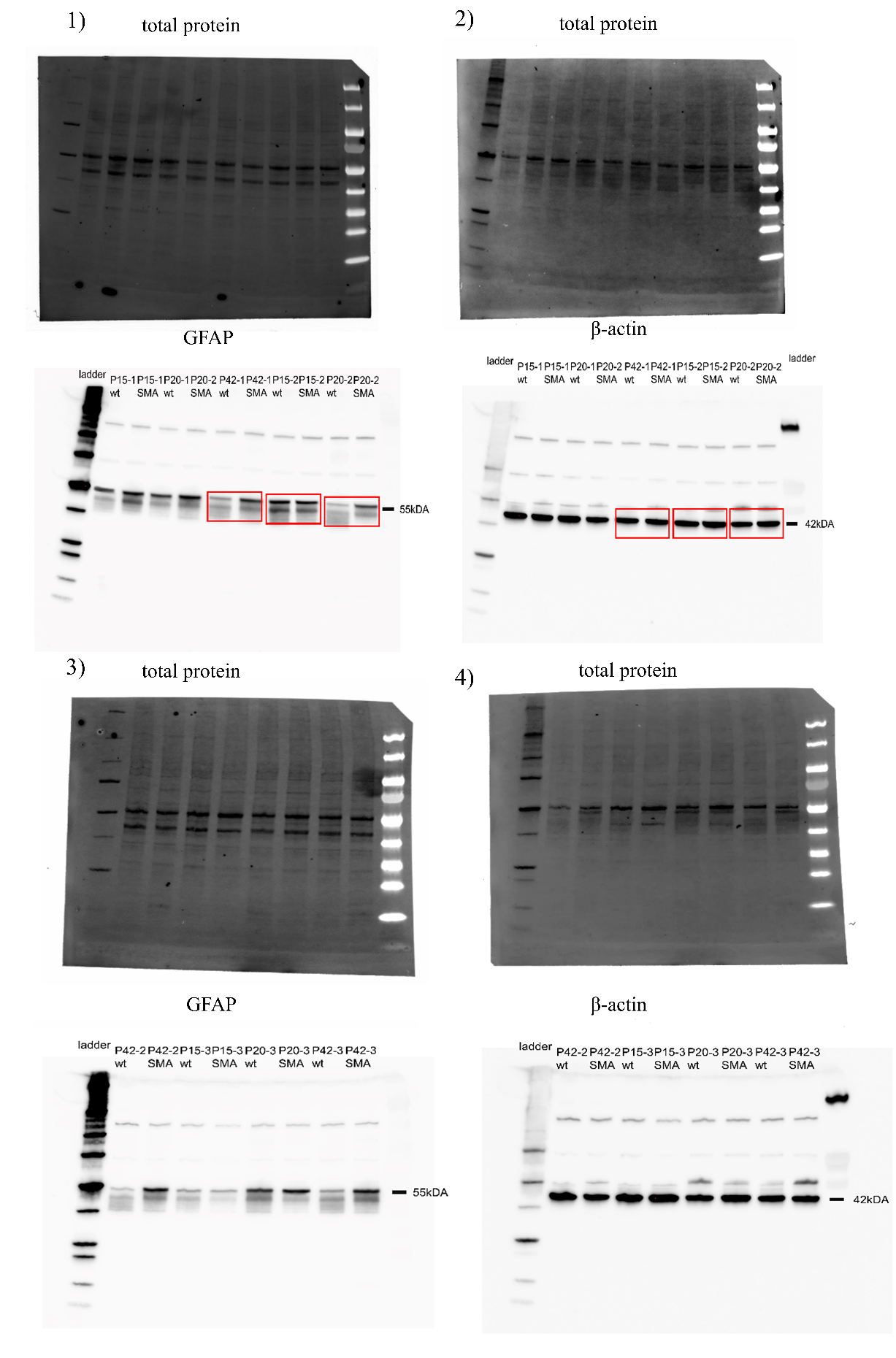


**Supplementary Figure 2:** Total protein staining of the membrane. Western Blot of GFAP and Actin of wild-type and late-onset SMA mice at P15, P20 and P42.

Western blot of wild-type and late-onset SMA mice (3 individual mice per condition). Total protein, EAAT1 (anti-rabbit Synaptic Systems) and Actin (anti-rabbit Abcam) staining; red box indicates used WB in the manuscript.


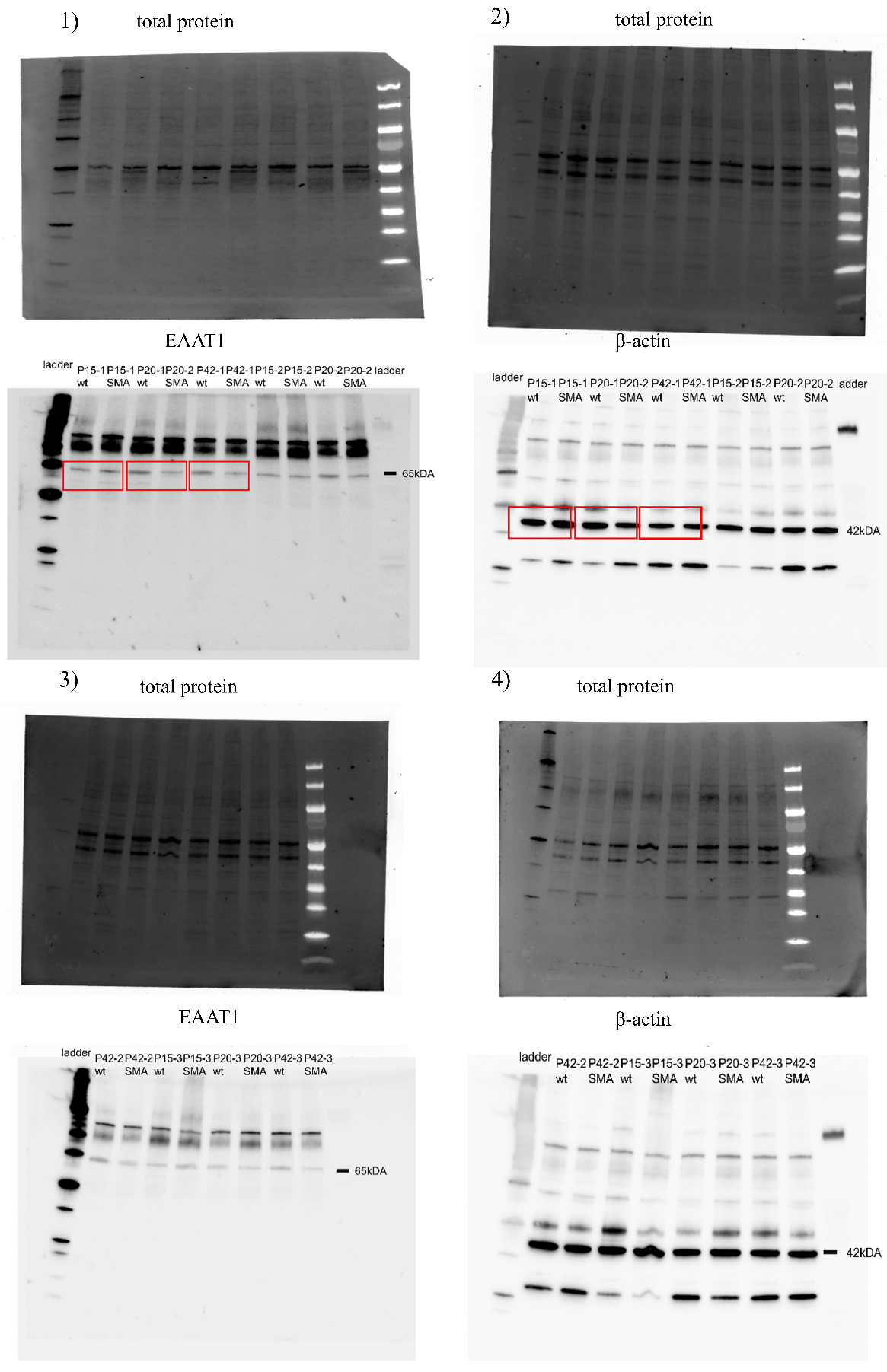


**Supplementary Figure 3:** Total protein staining of the membrane. Western Blot of EAAT1 and Actin of wild-type and late-onset SMA mice at P15, P20 and P42

Western blot of veh treated SMA mice. AA treated SMA mice and veh treated wild-type mice (3 individual mice per condition). Total protein, SMN (anti-rabbit Novus Biologicals) , GFAP (anti-rabbit Synaptic Systems) and Actin (anti-rabbit Abcam) staining; red box indicates used WB in the manuscript.


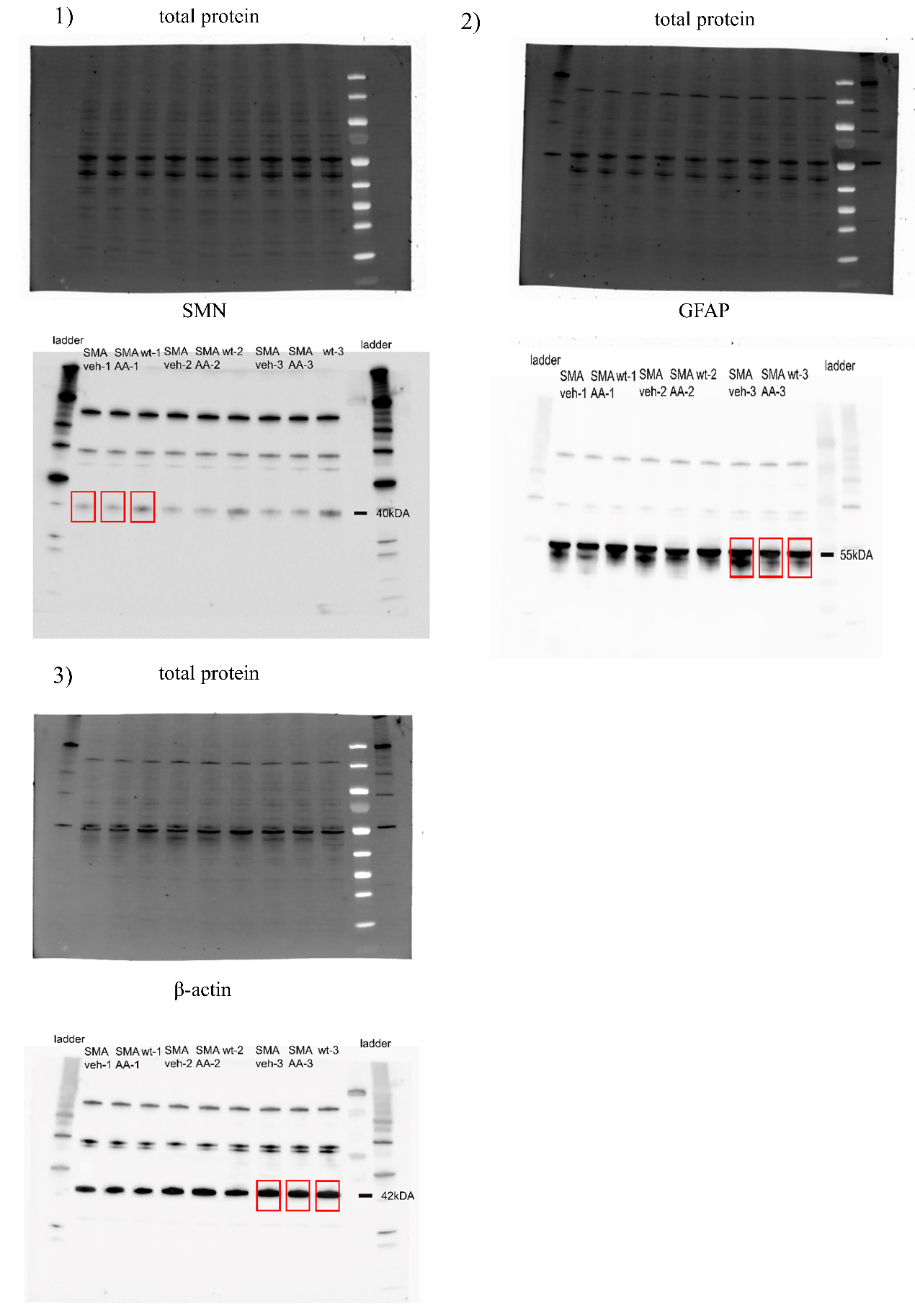


**Supplementary Figure 4:** Total protein staining of the membrane. Western Blot of SMN, GFAP and Actin of veh treated SMA mice, AA treated SMA mice and veh treated wild-type mice.

Western blot of veh treated SMA mice. AA treated SMA mice and veh treated wild-type mice (3 individual mice per condition). Total protein, EAAT1 (anti-rabbit Synaptic Systems) and Actin (anti-rabbit Abcam) staining; red box indicates used WB in the manuscript.


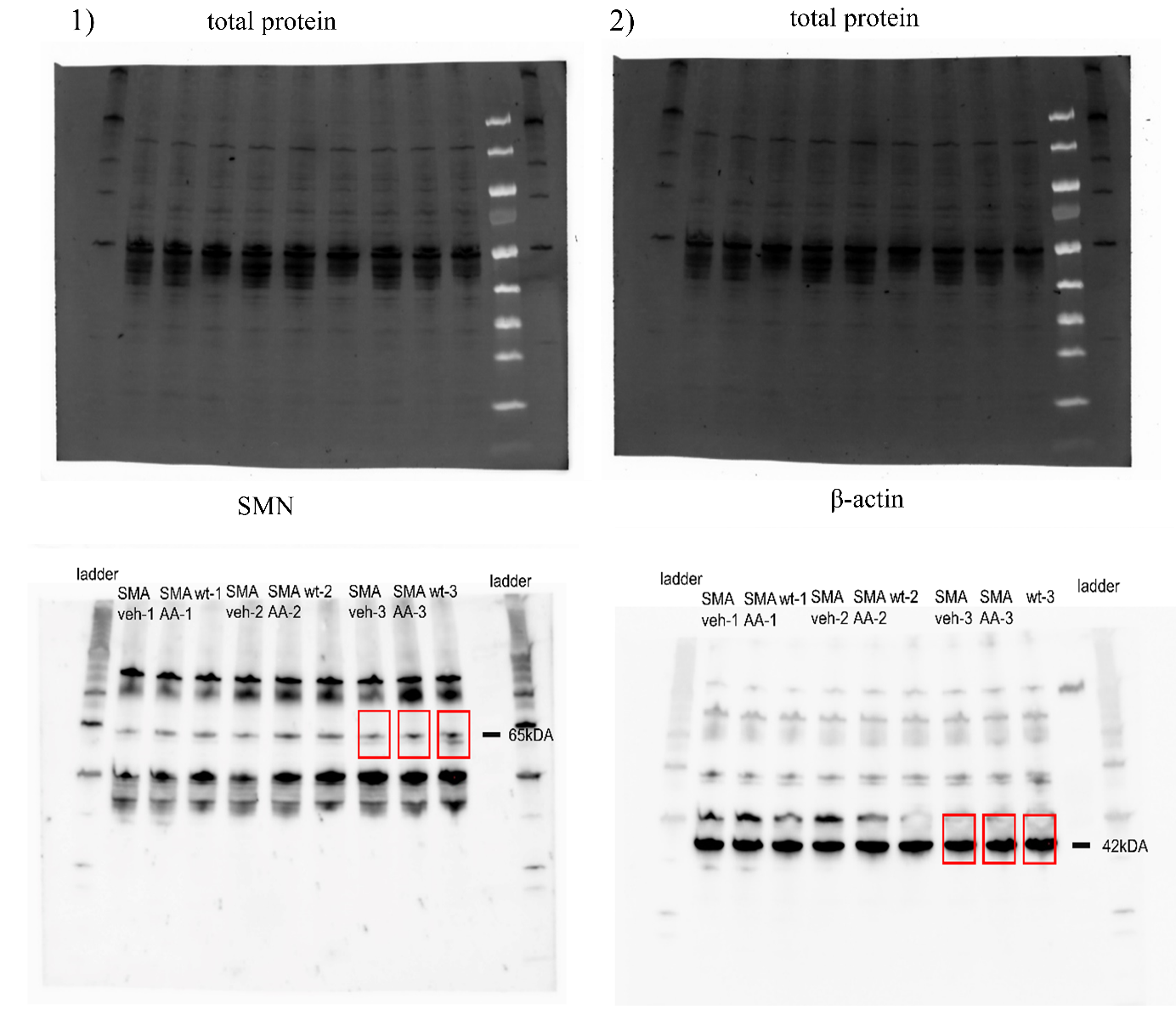


**Supplementary Figure 5:** Total protein staining of the membrane. Western Blot of EAAT1 and Actin of veh treated SMA mice, AA treated SMA mice and veh treated wild-type mice.

Western blot of control and SMN siRNA treated mice astrocytes (3 individual mice per condition). Total protein, EAAT1 (anti-rabbit Synaptic Systems) and Actin (anti-rabbit Abcam) staining; red box indicates used WB in the manuscript.


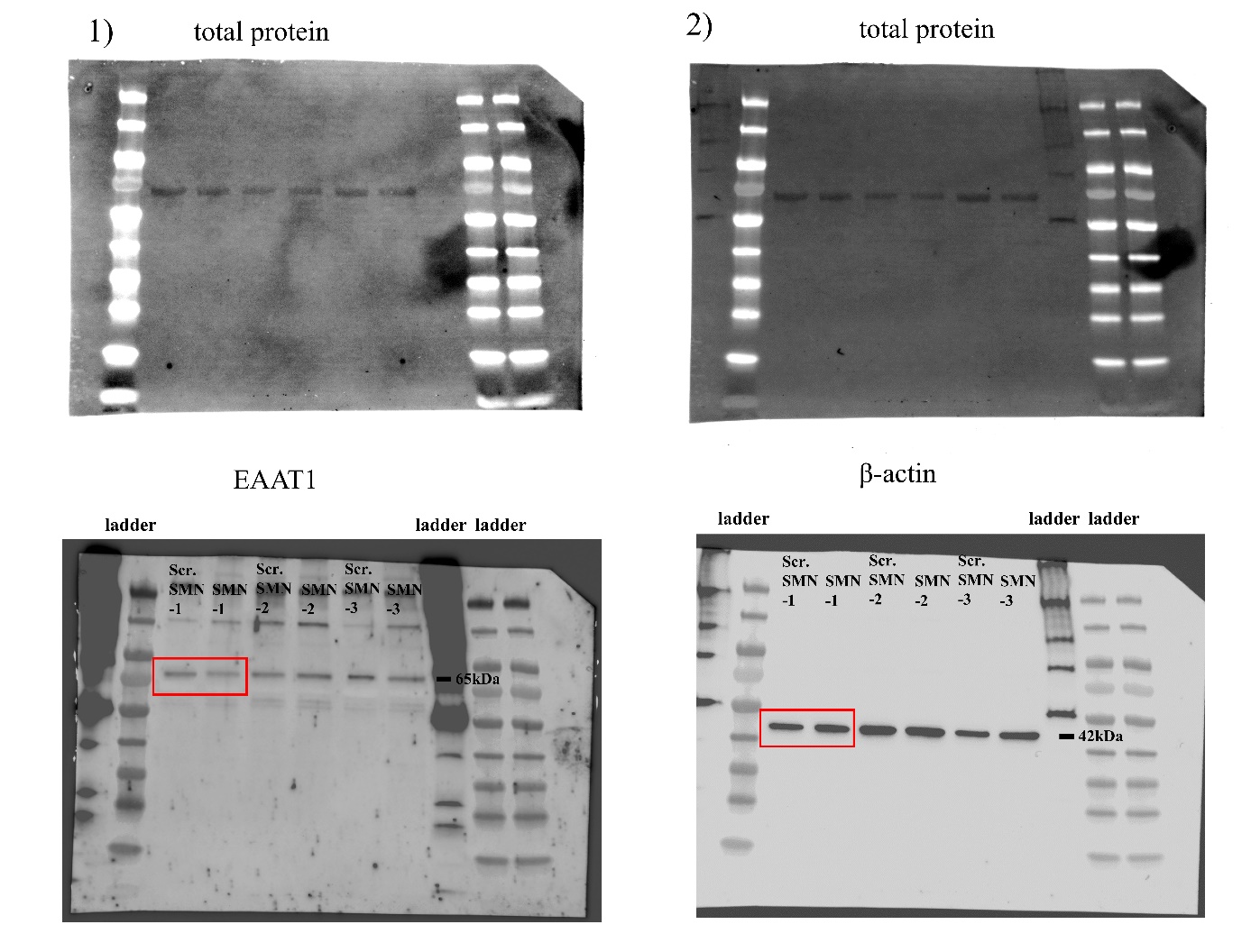


**Supplementary Figure 6:** Total protein staining of the membrane. Western Blot of EAAT1 and Actin of control and SMN siRNA treated mice astrocytes.

Immunohistochemistry of SMI-32 positive cells (motor neuron) in the ventral horn of the spinal cord at P15, P20 and P42 for wt and SMA mice.


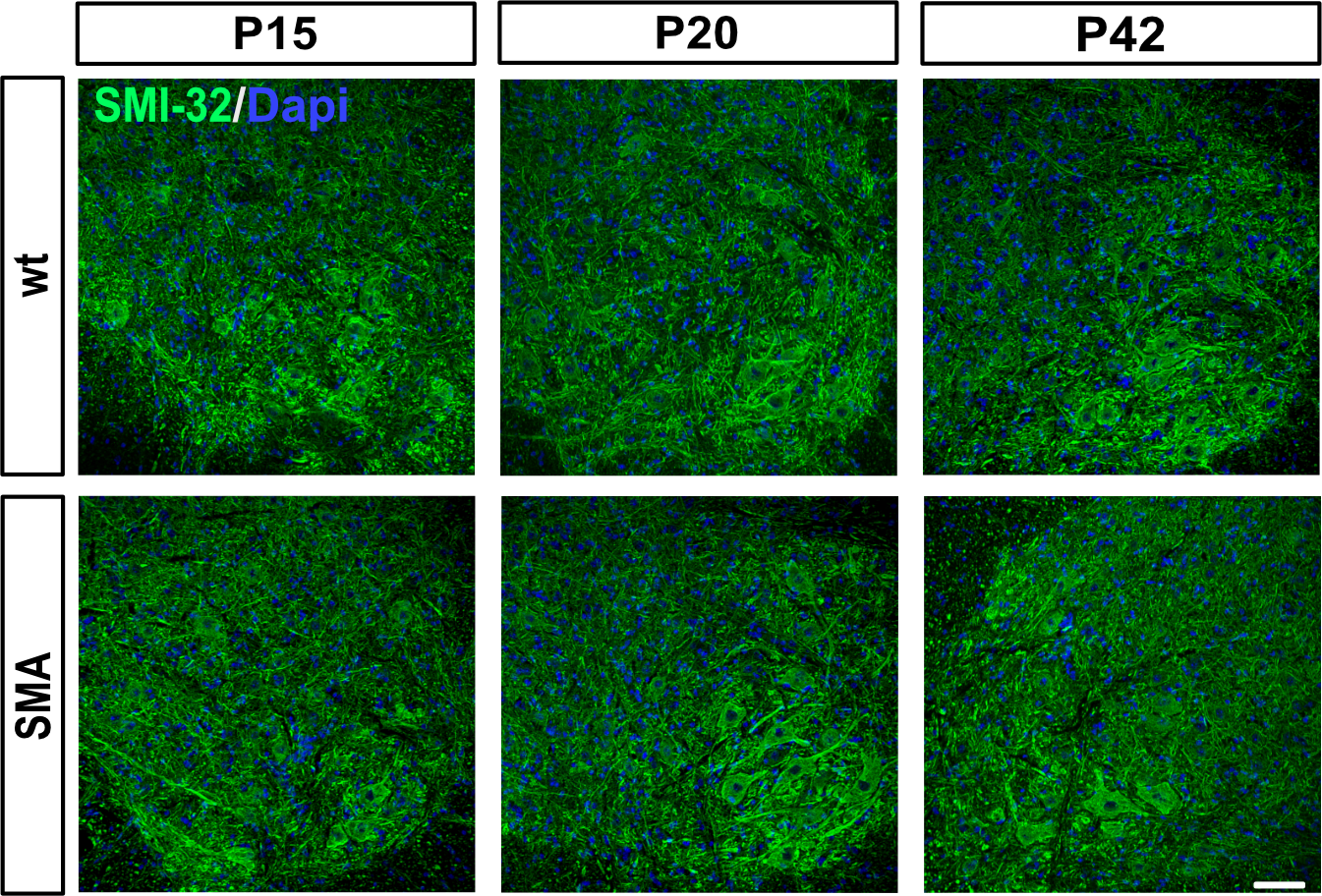


**Supplementary Figure 7:** SMI-32 staining for MN in the ventral horn of the spinal cord at P14, P20 and P42 for wt and SMA mice.

Immunohistochemistry of SMI-32 positive cells (motor neuron) in the ventral horn of the spinal cord at P44 containing SMA veh, SMA AA and wt veh mice.


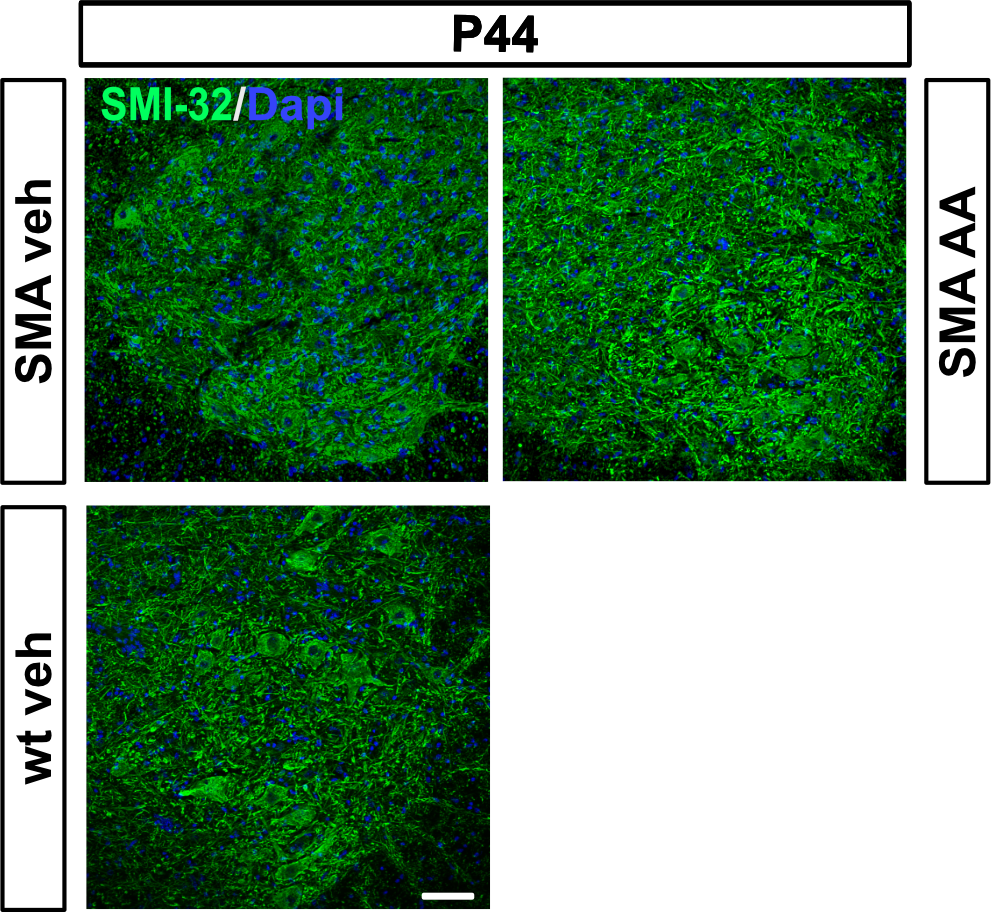


**Supplementary Figure 8:** SMI-32 staining for MN in the ventral horn of the spinal cord at P44 for SMA vehicle-treated, SMA AA-treated and wt vehicle-treated mice.

Table of used statistical analysis and test for normal distribution.

**Tab.1:** table of used statistical analysis and testing for normal distribution.

| Figure |  | Distribution | | Test |
| --- | --- | --- | --- | --- |
|  | | **normal** | **not normal** |  |
| 1 | a P33 | x |  | Welch’s T-test |
|  | a P44 | x |  | Welch’s T-test |
|  | b | x |  | Welch’s T-test |
|  | d | x |  | Welch’s T-test |
|  | e | x |  | Welch’s T-test |
| 2 | a | x |  | Welch’s T-test |
|  | b |  | x | Mann-Whitney test |
|  | c | x |  | Welch’s T-test |
| 3 | a | x |  | Welch’s T-test |
|  | b | x |  | Welch’s T-test |
|  | c | x |  | Welch’s T-test |
|  | d |  | x | Mann-Whitney test |
| 4 | a | x |  | Welch’s T-test |
|  | b |  | x | Mann-Whitney test |
|  | c |  | x | Mann-Whitney test |
|  | d | x |  | Welch’s T-test |
|  | e | x |  | Welch’s T-test |
|  | f | x |  | Ordinary one-way ANOVA |
|  | g | x |  | Welch’s T-test |
| 5 | a | x |  | Ordinary one-way ANOVA |
|  | b | x |  | Ordinary one-way ANOVA |
|  | c | x |  | Ordinary one-way ANOVA |
|  | d | x |  | Ordinary one-way ANOVA |
| 6 | a | x |  | Ordinary one-way ANOVA |
|  | b | x |  | Ordinary one-way ANOVA |
|  | c |  | x | Kruskal-Wallis test ANOVA |
|  | d | x |  | Ordinary one-way ANOVA |
|  | e |  | x | Kruskal-Wallis test ANOVA |
|  | f | x |  | Ordinary one-way ANOVA |
|  | g |  | x | Kruskal-Wallis test ANOVA |
| 7 | a |  | x | Kruskal-Wallis test ANOVA |
|  | b | x |  | Ordinary one-way ANOVA |
|  | c P33 | x |  | Ordinary one-way ANOVA |
|  | c P44 | x |  | Ordinary one-way ANOVA |
|  | d P33 | x |  | Ordinary one-way ANOVA |
|  | d P44 | x |  | Ordinary one-way ANOVA |
|  | e P33 | x |  | Ordinary one-way ANOVA |
|  | e P44 | x |  | Ordinary one-way ANOVA |
|  | f P33 | x |  | Ordinary one-way ANOVA |
|  | f P44 | x |  | Ordinary one-way ANOVA |
|  | g | x |  | Ordinary one-way ANOVA |
| 8 | a |  | x | Kruskal-Wallis test ANOVA |
|  | b |  | x | Kruskal-Wallis test ANOVA |
|  | c | x |  | Ordinary one-way ANOVA |
|  | e | x |  | Ordinary one-way ANOVA |
|  | f | x |  | Ordinary one-way ANOVA |
|  | g | x |  | Ordinary one-way ANOVA |
| 9 | a | x |  | Welch’s T-test |
|  | b | x |  | Welch’s T-test |
|  | c | x |  | Welch’s T-test |
|  | d | x |  | Welch’s T-test |
|  | e | x |  | Welch’s T-test |
|  | f | x |  | Welch’s T-test |
|  | g | x |  | Ordinary one-way ANOVA |
